# Deactivatable Bisubstrate Inhibitors of Protein Kinases

**DOI:** 10.3390/molecules27196689

**Published:** 2022-10-08

**Authors:** Tanel Sõrmus, Darja Lavogina, Erki Enkvist, Asko Uri, Kaido Viht

**Affiliations:** Institute of Chemistry, University of Tartu, 14A Ravila St., 50411 Tartu, Estonia

**Keywords:** protein kinase, deactivatable inhibitor, photocage, bivalent, bisubstrate, multivalent, stimuli-responsive

## Abstract

Bivalent ligands, including bisubstrate inhibitors, are conjugates of pharmacophores, which simultaneously target two binding sites of the biomolecule. Such structures offer attainable means for the development of compounds whose ability to bind to the biological target could be modulated by an external trigger. In the present work, two deactivatable bisubstrate inhibitors of basophilic protein kinases (PKs) were constructed by conjugating the pharmacophores via linkers that could be cleaved in response to external stimuli. The inhibitor ARC-2121 incorporated a photocleavable nitrodibenzofuran-comprising β-amino acid residue in the structure of the linker. The pharmacophores of the other deactivatable inhibitor ARC-2194 were conjugated via reduction-cleavable disulfide bond. The disassembly of the inhibitors was monitored by HPLC-MS. The affinity and inhibitory potency of the inhibitors toward cAMP-dependent PK (PKAcα) were established by an equilibrium competitive displacement assay and enzyme activity assay, respectively. The deactivatable inhibitors possessed remarkably high 1–2-picomolar affinity toward PKAcα. Irradiation of ARC-2121 with 365 nm UV radiation led to reaction products possessing a 30-fold reduced affinity. The chemical reduction of ARC-2194 resulted in the decrease of affinity of over four orders of magnitude. The deactivatable inhibitors of PKs are valuable tools for the temporal inhibition or capture of these pharmacologically important enzymes.

## 1. Introduction

Ligands whose binding to the target biomolecule can be altered by spontaneous changes of microenvironment or on-demand by application of external chemical or physical triggers are indispensable tools in many fields, spanning from basic biochemical research to the development of biomedical and agricultural applications. The dynamic bioactivity can be implemented by equipping ligands with stimuli-responsive moieties. Ideally, stimuli-responsiveness enables binary on/off control over the affinity of the ligand. In practice, transition between the low-affinity (corresponding to the deactivated or ‘off-state’) and the high-affinity (active or ‘on-state’) form of the ligand occurs.

The applicable stimuli cover a broad range of signals, including changes of pH and redox potential, irradiation, exposure to enzymatic activity or reactive oxygen species, etc. [1]. Ultraviolet or visible light as the stimulus is a unique tool because it can be applied remotely, focused, and is easily adjustable in terms of wavelength, intensity, and duration of exposure. This not only provides the means for altering the bioactivity at the spatiotemporally focused point, but also allows gradual transformation of the bioactive compound by regulating the amount of energy applied to the system. Most photo-responsive ligands belong to two categories: (1) photocaged or photoactivatable ligands that undergo irreversible activation [2,3] and (2) photoswitchable ligands that can be toggled between the active and deactivated form [4]. A third category involves ligands that can be (3) irreversibly deactivated by UV-Vis irradiation.

Photocaging of small molecules is relatively straightforward, especially when the key functional groups (hot spots) of the ligand-target interaction have been established. Photoswitchable and irreversibly photo-deactivatable ligands represent alternative means to photocaged biological targets (e.g., proteins) for regulation of biological processes. However, the design of those compounds is more challenging, because embedding the responsive moiety into the structure of the ligand has to be tolerated by the other interaction partner. The development of photoswitchable ligands is an intensive area of research which has been extensively reviewed [4]. The principles for constructing irreversibly deactivatable and photoswitchable ligands are similar. Irreversibly deactivatable ligands could be developed by replacing a part of the pharmacophore with a cleavable moiety. The latter structural units can be bioisosteric to the original fragment (for example: [5]) or block functional groups unfavorable for the complex formation [6]. This approach is limited by the choice of stimuli-responsive bioisosteres and it poses a risk of affinity loss. Another approach is to embed the cleavable moiety to a position that is less critical for assembling the pharmacophore, e.g., in the internal positions of the interacting sequences of peptides [7,8,9,10], oligonucleotides [11,12,13], and even proteins [14]. Photolysis disassembles such compounds into low-affinity fragments, resulting in loss of bioactivity. The same principle has been used for the construction of chelate moieties of photocaged metal ions, e.g., Ca^2+^ [15,16], and photocleavable bivalent ligands.

**Figure 1:**
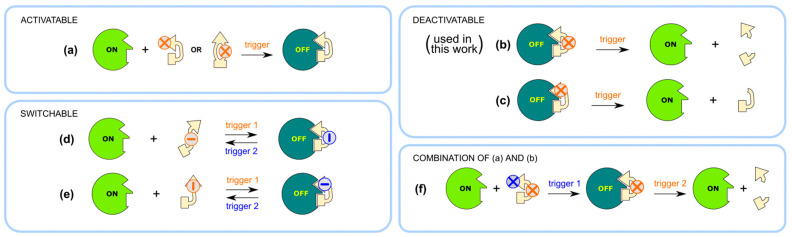
Approaches for the development of bivalent ligands with dynamic bioactivity. (**a**) activatable ligand is constructed by blocking a single fragment of the bivalent ligand by a cleavable group or altering the spatial organization of the fragments by cyclization of the bivalent ligand via a cleavable linker; (**b**,**c**) irreversibly deactivatable ligand is made by incorporating a cleavable linker (**b**) or a cleavable moiety as one of the pharmacophores (**c**); (**d**,**e**) switchable ligand is made by incorporating a switchable moiety into the linker (**d**) or as one of the pharmacophores I; (**f**) incorporating two fragments, which are cleavable via different triggers; applying one trigger results in compound activation, and applying the other trigger results in deactivation.

Bivalent ligands can be defined as conjugates of two pharmacophores, which simultaneously target two distinct binding sites of the same biomolecule. The affinity (or avidity) of the representatives of this class, i.e., bitopic ligands [17], bisubstrate inhibitors [18], etc., is generally higher if compared to the single site-targeting ligands which correspond to the same fragments. The free energy change of the binding of a bivalent ligand A–B to its receptor can be expressed as a sum of the binding energies of the individual fragments A and B plus a third term, which summarizes the factors arising from linking the fragments (Equation (1)) [19]:

(1) $$\Delta G_{A-B}^{}=\Delta G_{A}^{}+\Delta G_{B}^{}+\Delta G_{\mathrm{linking}}=RT\;\ln K_{D},\;$$

where *K*_D_ is the dissociation constant of the ligand-receptor complex. In essence, once one fragment is bound to its binding site, the other fragment is forced to proximity of its cognate binding site, which increases the probability of (re)binding of the latter fragment to the protein (chelate effect) [20]. As discussed previously, the additivity of the binding free energies of the fragments could result in decrease of *K*_D_ values by several orders of magnitude. However, the extent of the binding energy gain depends on several factors and is highly variable [18,21]. Importantly, it is possible to make ligands whose bivalent binding is under the control of stimuli-responsive moieties. In addition, as each fragment has a smaller individual contribution to the overall binding energy, bivalent ligands could also be more amenable to structural changes, such as the incorporation of stimuli-responsive moieties. The modularity of bivalent construction gives several possibilities for designing ligands whose ability to bind to the target could be enhanced or suppressed by an external trigger. Moreover, these functions could be implemented by incorporating a single responsive moiety, which is important for minimizing the structural complexity of the ligand (Figure 1).

An example of a photocaged bisubstrate inhibitor (Figure 1a) of protein kinases (PKs) was recently published by our group [22]. PKs are transferases that catalyze protein phosphorylation, a key reaction of regulation of cell’s life. PKs are also important drug targets as aberrations in protein phosphorylation balances are associated with different diseases, including cancer [23]. The bisubstrate inhibitors of PKs developed in our group, termed ARCs, comprise two inhibitory domains: an aromatic moiety, which is targeted to the ATP-binding site of the PK, and a peptide or peptide analogue, which is targeted to the protein substrate-binding site of PK. In the case of ARCs targeting basophilic PKs, the peptide part typically comprises a cluster of d-arginine residues. These inhibitory domains are joined via a linker that enables simultaneous association of the domains with the aforementioned binding regions of the PK. The conjugation of the fragments yields inhibitors with a remarkable potency, where the lowest *K*_D_ values of ARCs are in the low picomolar range. The attachment of a single nitrodibenzofuran (NDBF) photocage to a hot-spot position on the ATP-binding site-targeting fragment efficiently blocked the binding of ARC to the catalytic subunit type α of cAMP-dependent PK (PKAcα), as shown by the five orders of magnitude affinity difference between the photocaged (*K*_D_ = 1.9 µM) and active (*K*_D_ = 5 pM) forms of the inhibitor [22]. Furthermore, several examples of bivalent (and multivalent) ligands have been published whose multivalent binding is controlled by a photoswitch (Figure 1d) [24,25,26,27,28].

The structure of bivalent ligands is also well suitable for the construction of deactivatable bioactive compounds by the ‘cleavable linker approach’. Inserting a cleavable moiety in the linker region of a bivalent ligand allows intentional decomposition of the ligand into two low-affinity fragments, thus reversing the principle of affinity gain of bivalent ligands (Figure 1b). This approach was used for the development of a photo-deactivatable bivalent inhibitor that simultaneously associated with the SH1 and SH2 domains of Src PK [29]. Photolysis of the most potent compound (IC_50_ = 18 nM) restored up to 90% of the activity of the kinase. In another study, a photo-deactivatable bifunctional inhibitor of pre-miRNA:Dicer complex was developed for light-mediated spatiotemporal control of miRNA maturation [30].

In the present work, the ‘cleavable linker approach’ was used to develop deactivatable ARC-type bisubstrate inhibitors of basophilic PKs. To the best of our knowledge, this approach has not been previously used for development of deactivatable bisubstrate inhibitors of PKs. Two deactivatable ARCs were constructed, one containing a photo-degradable linker, the other a disulfide bond-containing linker. Depending on the type of the linker, irradiation at 365 nm or chemical reduction of the complex of the ARC with the catalytic subunit of PKAcα decomposed the ARC into low-affinity fragments.

## 2. Results

### 2.1. Design and Synthesis of Deactivatable Bisubstrate Inhibitors of PKAcα

The linker connecting two pharmacophores of a bisubstrate inhibitor should be of suitable length and flexibility to afford the appropriate spatial positioning of the pharmacophores for simultaneous association with the target biomolecule. The substitution of a cleavable moiety for the non-cleavable linker is conceptually a simple approach for the development of deactivatable bisubstrate inhibitors. However, the linker itself may develop specific interactions important for the complex formation, which should be considered when modifying the structure.

**Figure 2:**
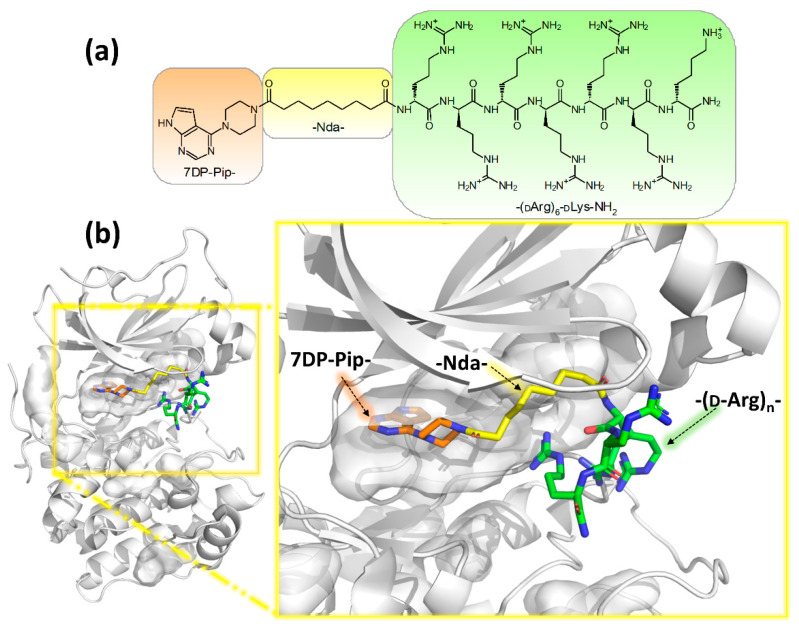
The structure and binding mode of ARC-1411 to PKAcα. (**a**) The structure of ARC-1411; (**b**) The co-crystal structure of ARC-1411:PKAcα complex (pdb: 5IZJ [31]) where only a part of the hexa-d-arginine is shown. PKAcα is shown as grey cartoon; the structure elements of ARC-1411 are shown with different colors: 7DP-Pip residue in the ATP-binding site as orange sticks, Nda linker as yellow sticks, and d-arginine residues as green sticks.

In the present work, two ARC-type bisubstrate inhibitors of basophilic PKs were constructed that could be deactivated either by applying a reducing agent or by irradiation at 365 nm. PKAcα was chosen as the target PK for demonstrating the deactivation of the inhibitors for the following reasons. PKAcα has been considered a prototype PK for which many crystal structures are available, including co-crystal structures of many PKAcα:ARC-type inhibitor complexes. Here, the design of the deactivatable inhibitors proceeded from the structure of a previously published inhibitor ARC-1411 that displays exceptionally high affinity towards PKAcα (*K*_D_ = 3 pM) and other basophilic PKs [31]. ARC-1411 (Figure 2a) is a conjugate of 7-deazapurine-piperazine pair (7DP-Pip) and hexa-d-arginine that are connected via a non-polar unbranched flexible aliphatic linker represented by nonanedioic acid residue (Nda). ARC-1411 also possesses a C-terminal d-lysine residue for the attachment of fluorescent labels [31]. The aforementioned building blocks are connected via amide bonds. A previously published co-crystal structure of ARC-1411:PKAcα complex (Figure 2b) demonstrates the bisubstrate binding mode of the inhibitor. Expectedly, 7DP-Pip-residue is fixed inside the ATP-binding cavity that is located between the N- and C-terminal lobes of PKAcα. The hexa-d-arginine peptide interacts with amino acid residues of the solvent-exposed substrate protein-binding region of PKAcα and is not fully covered by electron density, which points to a larger conformational freedom of the C-terminal part of the inhibitor in the complex. The linker part of ARC-1411 is relatively fixed and forms a curvature underneath the glycine-rich loop where it develops multiple interactions with PKAcα. In conclusion, the linker is an integral part of the inhibitor that undergoes specific conformational changes and forms interactions important for the association with the PK. Even subtle changes in the structure of the linker of an ARC-type inhibitor may have a remarkable effect on the affinity of the inhibitor.

**Scheme 1:**
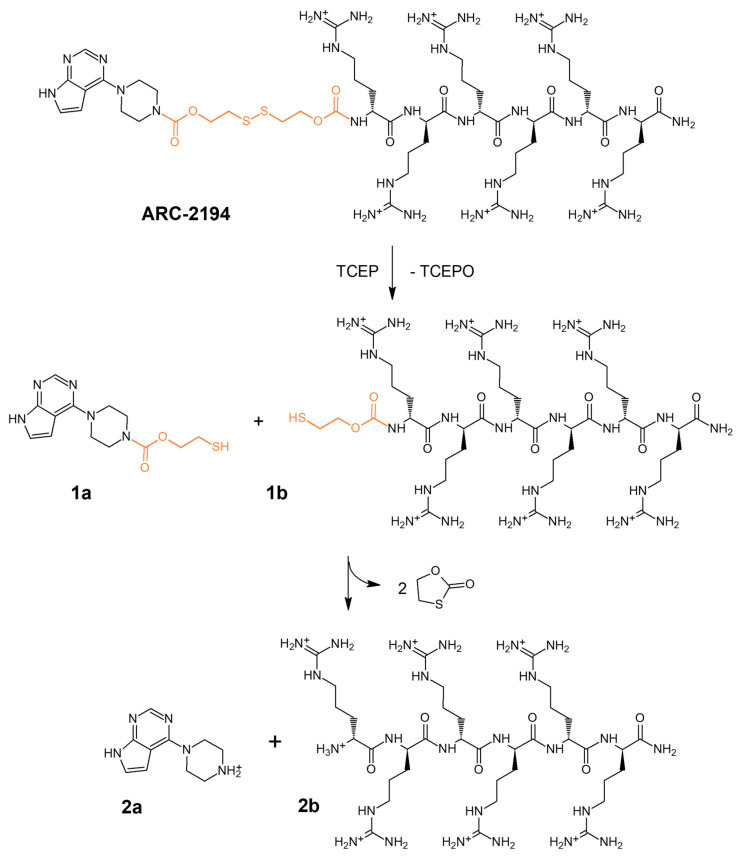
Chemical reduction of ARC-2194.

In the present work, it was assumed that PKAcα tolerates the replacement of Nda residue by a linear chain of similar length that incorporates disulfide junction. A novel redox-cleavable compound ARC-2194 was developed, which comprised a self-immolative disulfide linker [32] between 7DP-Pip-residue and hexa-d-arginine moiety. Differently from ARC-1411, the linker was connected to the structure via carbamate groups. The reductive cleavage of the disulfide bond of ARC-2194 was expected to liberate compounds **1a** and **1b**, which would self-disintegrate into final products **2a** (7DP-Pip) and **2b** (hexa-d-arginine-amide; Scheme 1). Both products were supposed to be very weak inhibitors of PKAcα (the analogues of **2a** [33] and **2b** [34] displayed IC_50_ values in the micromolar and millimolar range, respectively).

The replacement of the Nda linker in the structure of ARC-1411 with a photocleavable group was not considered for steric reasons. Instead, photocleavable chiral β-amino acid (compound **3**) was designed and introduced between the linker and the oligo-d-arginine moiety. This substitution was based on the previous knowledge that in this position chiral amino acids in d-configuration are strongly preferred for good binding by PKAcα whereas the side chain of this amino acid may be variable and even accommodate bulky substituents [35]. It was expected that **3** minimally changes the backbone of the inhibitor and that the bulky photosensitive aromatic structure forming the side chain of the photocleavable β-amino acid would be oriented towards the solution phase and thus not cause steric hindrance when the inhibitor associates with PKAcα.

**Scheme 2:**
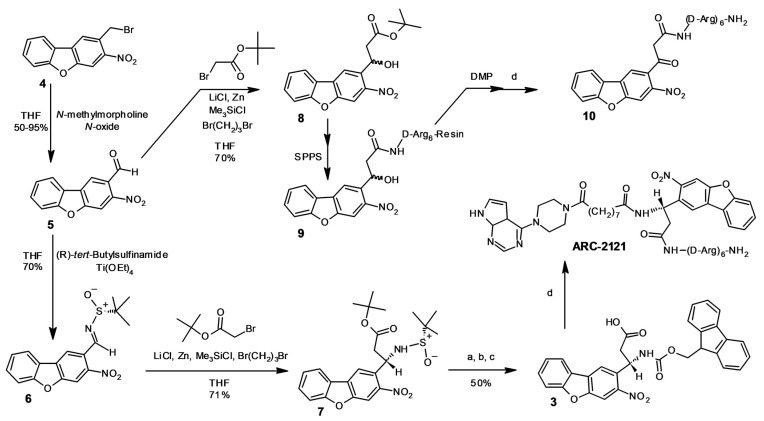
The chemical synthesis of ARC-2121 and compound **10**. (**a**) HCl/EtOAc; Et_2_O: dioxane (rt, 35 min); (**b**) Fmoc-Osu, TEA; CH_3_OH: CH_2_Cl_2_ (rt, overnight); (**c**) TFA: H_2_O: TES (rt, 3 h); (**d**). Solid phase peptide synthesis (SPPS), followed by cleavage from the resin and HPLC purification. THF—tetrahydrofuran; DMP—Dess-Martin periodinane; Fmoc-Osu—*N*-(9-Fluorenylmethoxycarbonyloxy)succinimide.

The side chain of **3** was composed of the photochemically active NDBF group [15]. NDBF was chosen to the role of the photo-degradable functionality as it possesses high photolysis quantum yield (*φ* = 0.7) and extinction coefficient (*ε*_330 nm_ = 18,400 M^−1^ cm^−1^) which in turn brings about high photolysis efficacy (*εφ* = 12,880 M^−1^ cm^−1^), higher than these of the commonly used nitrobenzyl-based photocleavable groups. For the construction of compound **3**, NDBF-β-amino acid with defined chirality, an enantioselective synthesis route (Scheme 2) was developed that was based on a previously published general method for enantioselective synthesis of β-amino esters via chiral *N*-*tert*-butanesulfinyl imines [36]. The synthesis started from 2-bromomethyl-3-nitrodibenzo[*b*,*d*]furan (compound **4**) [22] that was oxidized to an aldehyde (compound **5**) [37], which was then transformed into an enantiomer of *N*-*tert*-butanesulfinyl imine (compound **6**). The latter compound was coupled with Boc-protected carboxylic acid by enantioselective aza-Reformatsky reaction (compound **7**) [36]. Finally, the Boc and Boc-sulfinamide protecting groups were removed in acidic conditions and the resulting β-amino acid was Fmoc-protected (compound **3**) for use in solid phase peptide synthesis (SPPS) of the novel photocleavable inhibitor ARC-2121 (Scheme 2). The reactions with NDBF derivates were performed in the dark to prevent premature photolysis.

**Scheme 3:**
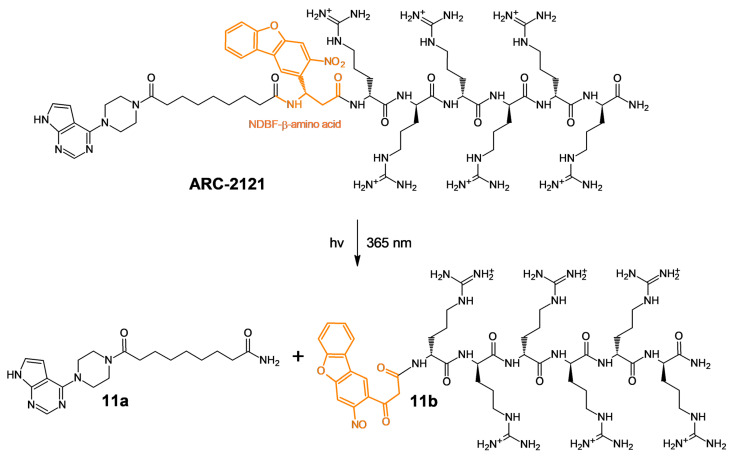
Reaction scheme of photo-fragmentation of ARC-2121, according to the reported main pathway of the NBDF group photolysis [3].

According to the proposed main route [3], the photo-degradation of ARC-2121 (Scheme 3) led to two main products: 7DP-Pip-Nda-amide (compound **11a,** ARC-2104) and a derivative of hexa-d-arginine-amide, which had nitrosodibenzofuranyl group attached to the N-terminus via malonic acid residue (compound **11b**). Compounds **11a** and **10** (ARC-2167), an analogue of **11b** (Scheme 2), were synthesized as reference compounds.

### 2.2. Characterization of Deactivatable Inhibitors

Before application of the trigger, the deactivatable bisubstrate inhibitor is a single chemical entity whose affinity can be characterized by the dissociation constant (*K*_D_) of the complex with the target enzyme. After applying the trigger, the inhibitor is disassembled into low-affinity fragments, which determine the post-trigger affinity. However, if the transition is not quantitative, the maximal change of affinity is not realized. The post-trigger affinity could be characterized by an IC_50_ value or an apparent dissociation constant (*K*_D_^app^) that corresponds to the mixture obtained after transformation. The calculations of *K*_D_^app^ are based on the concentration of the inhibitor before applying the trigger.

In the present work, photoluminescence-based competitive equilibrium binding assay was used to establish the *K*_D_ and *K*_D_^app^ values of the novel compounds as determined before and after fragmentation, respectively. The compounds displaced photoluminescent probes from the complex with PKAcα in a concentration dependent manner, which resulted in a decrease of the output signal of the assay. Two assay formats were used, which were designed for the determination of *K*_D_ values of high-affinity bivalent inhibitors and the mixtures of their low-affinity fragments, respectively.

The first assay was based on the measurement of the time-gated photoluminescence signal intensity (TGLI) and it utilized ARC-Lum(Fluo) probes ARC-1063 or ARC-1182, which both emit long life-time photoluminescence signal when associated with a target PK [38]. ARC-1063 and ARC-1182 differ by the fluorescent label (Figure S7) and the choice between these probes was only based on the availability of the particular probe. The integrated TGLI between 60–260 µs after the flash excitation at 337 nm correlates with the concentration of ARC-Lum(Fluo):PKAcα complex in the solution. The stability of ARC-1063:PKAcα and ARC-1182:PKAcα complexes is very high (*K*_D_^*^ = 15 pM [39]; the dissociation equilibrium constants of photoluminescent probes are designated as *K*_D_^*^ to distinguish them from the *K*_D_ values of the investigated compounds). Since the output TGL signal is only emitted by the ARC-Lum(Fluo):PKAcα complex and not by free photoluminescent probe, ARC-Lum(Fluo) can be used in excess if compared to the concentration of PKAcα (in the present work, 10 nM ARC-1063 or 10 nM ARC-1182 and 1 nM PKAcα were used). The combination of these factors increases the IC_50_ values of the competing ligands and a linear relationship between the log*K*_D_ and logIC_50_ is observed already in the picomolar range of *K*_D_ values (Figure S1). Thus, this assay configuration can be used for determination of *K*_D_ values of high affinity inhibitors. However, low-affinity compounds will show too high IC_50_ values that may be out of the reasonable concentration range of the compounds.

The second assay was based on measuring the change of fluorescence anisotropy (FA, Δ*r*) of the photoluminescent probe ARC-583 (*K*_D_^*^ = 0.5 nM [40], Figure S7). A typical assay setup based on the measurement of FA utilizes concentrations of the fluorescence probes slightly below the concentration of the target protein but higher than the *K*_D_^*^ to achieve maximal Δ*r*. The assay in the present work was conducted at 2 nM ARC-583 and 3 nM PKAcα. In these conditions, the logIC_50_ is linearly dependent on the log*K*_D_ above 1 nM *K*_D_ value (Figure S1). This assay cannot be used for characterizing inhibitors with picomolar *K*_D_ values: these inhibitors would demonstrate the tight-binding character (i.e., large contribution of the concentration of the enzyme to the observed IC_50_ value because of inhibitor depletion). However, the IC_50_ values of the competing compounds above the tight binding limit are smaller than the IC_50_ values of the same compounds observed by the TGLI-based assay. Thus, the *K*_D_ values for low-affinity compounds can be reliably established with this FA-based assay format.

H-89, a non-selective inhibitor of PKAcα (Figure S7), was used as a reference compound because the IC_50_ value for this compound was expected to be within the measurable range of both assay systems (*K*_D_ of the H-89:PKAcα complex is 9 nM [41]). The displacement data were fitted to the previously described [40,42] exact model of competitive binding (further explained in Appendix A) to determine *K*_D_ and *K*_D_^app^ values, which were used as concentration- and probe-independent quantities to evaluate the deactivation efficiencies of the novel compounds.

**Figure 3:**
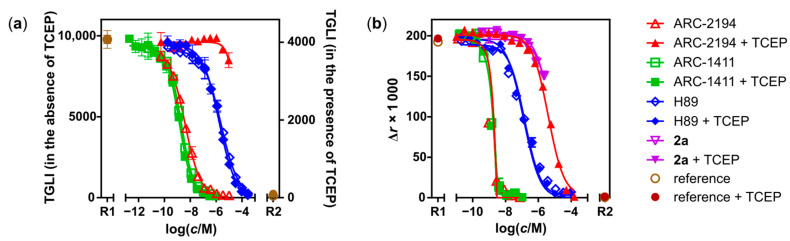
Displacement assays of the redox-sensitive ARC-2194 and reference compounds. Displacement of photoluminescent probes (**a**) ARC-1063 and (**b**) ARC-583 from the complex with PKAcα by ARC-1411 (green rectangles), ARC-2194 (red triangles), H-89 (blue rhombi) and **2a** (purple triangles, only on panel (**b**)), all in the absence (open symbols) and in the presence (closed symbols) of TCEP (1 mM). The displacement was followed by (**a**) the change of TGLI signal corresponding to ARC-1063:PKAcα complex and (**b**) by the change of anisotropy of fluorescence emitted by ARC-583. Reference solutions (brown symbols) contained the corresponding photoluminescent probes in the presence (R1) or in the absence (R2) of PKAcα. The reported values represent the means ± SD from two parallel experiments.

First, the redox-cleavable inhibitor ARC-2194 was characterized by the competitive equilibrium binding assay. The lead compound, ARC-1411, and the expected fragmentation product **2a** were used as reference inhibitors. The displacement curves for all compounds were measured before and after addition of the reducing agent tris(2-carboxyethyl)phosphine (TCEP, Figure 3 and Table 1). As expected, ARC-1411 showed very steep displacement curve if measured in the FA-assay. The obtained result points to the tight-binding character of the inhibitor. Therefore, this curve was not used for the *K*_D_ determination. However, the TGLI assay afforded the *K*_D_ value for ARC-1411:PKAcα complex (*K*_D_ = 3 ± 1 pM) that was in agreement with the previously measured affinity (Table 1). The *K*_D_ value of ARC-2194 (7 ± 1 pM) could be established only by the TGLI assay and it was just marginally higher than the *K*_D_ value of ARC-1411, which demonstrated that the structural modification leading to ARC-2194 were well tolerated by PKAcα (slightly longer linker, which comprises a disulfide bond, replacement of amide bonds with carbamate groups). **2a** was only characterized in the FA assay, which revealed its affinity for PKAcα to be in the sub-micromolar range.

**Figure 4:**
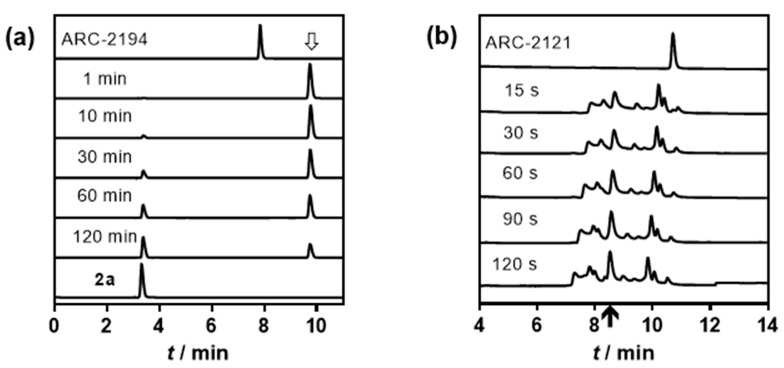
Inhibitor deactivation monitored with HPLC. (**a**) HPLC monitoring of the cleavage of ARC-2194 (100 µM) by TCEP (1 mM; pH = 7.5, 30 °C). The top and bottom trace correspond to reference compounds ARC-2194 and **2a**, as indicated. The peak at *R*_t_ = 9.74 min (indicated by the white arrow) corresponds to compound **1a**. (**b**) HPLC monitoring of the photo-fragmentation of ARC-2121 (200 μM). The top trace corresponds to intact ARC-2121. The peak at *R*_t_ = 8.52 min (indicated by the black arrow) corresponds to **11a**. The same elution gradient was used in **a** and **b**, and the traces correspond to absorbance changes at 290 nm.

The ARC-583-based FA assay well tolerated the addition of TCEP to the measurement solution. The displacement curves of **2a**, ARC-1411, and H-89 were not influenced by TCEP, which proves that the affinities of all these compounds, including ARC-583 itself as well as the optical properties of TAMRA-dye attached to ARC-583, were not affected by TCEP. Upon TCEP addition to the TGLI assay mixtures, 2.5-fold decrease of TGLI was observed. This can be explained by the reversible addition of TCEP to the cyanine dye Alexa Fluor-647 (a fluorescent label of ARC-1063) that is known to cause reversible quenching of fluorescence [43]. However, the normalized displacement curves of ARC-1411 and H-89 before and after TCEP treatment, respectively, coincide (Figure 3), which proves that only the TGLI and not the affinity of ARC-1063 was affected by TCEP. The *K*_D_^app^ calculated from the displacement curve of ARC-2194 after TCEP-treatment was by 4.5 orders of magnitude higher than the *K*_D_ value of untreated ARC-2194 and it was practically identical to the *K*_D_ value of **2a**, one of the fragmentation products. This shows that the inhibitor was quantitatively disassembled in the used conditions. This conclusion was also consistent with the HPLC-MS monitoring of the cleavage reaction (Figure 4). As demonstrated by others [44], addition of an excess amount of TCEP rapidly reduced the disulfide bond. Within less than 1 min, the parent compound ARC-2194 had been quantitatively converted to **1a**, followed by a slower decomposition of the latter to **2a** (Figure S6). After 2 h, more than 50% of **1a** had been converted to **2a**. However, during this timeframe, the time-dependent shift of displacement curves was not observed, showing that the IC_50_ values of **2a** and **1a** were in the same concentration region and the full effect of deactivation of ARC-2194 was already realized after the first reductive cleavage step.

**Figure 5:**
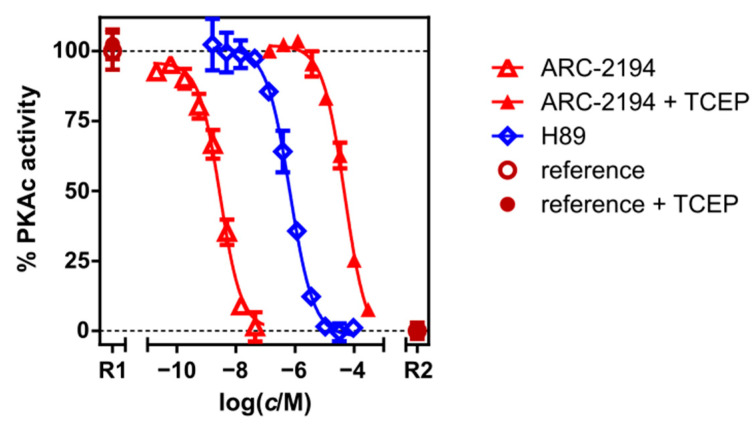
Inhibition of the catalytic activity of PKAcα by ARC-2194 (red triangles) and H89 (blue rhombi). Reference solutions (brown spheres) correspond to: (R1) the phosphorylation mixtures (1 mM MgATP, 10 µM peptide substrate, 0.5 nM PKAcα) in the absence of inhibitors, and (R2) the same in the absence of PKAcα. The open and closed symbols correspond to the absence and to the presence of TCEP (1 mM) in the mixtures, respectively. The reported values represent the means ± SD from two parallel experiments.

The inhibitory potency of ARC-2194 was assayed by real time monitoring of the PKAcα-catalyzed phosphorylation reaction in the presence of the inhibitor (Figure 5). At 1 mM ATP, the IC_50_ of ARC-2194 was 2.9 nM, which is in accordance with the *K*_D_ value in the picomolar range (Table 1). The reference compound H89 showed submicromolar inhibitory potency in these conditions. Upon co-incubation with TCEP, an IC_50_ value 4.2 orders of magnitude higher for ARC-2194 was registered, which demonstrates that the deactivation of the inhibitor could be followed both by the competitive displacement assay and enzyme kinetic assay.

**Figure 6:**
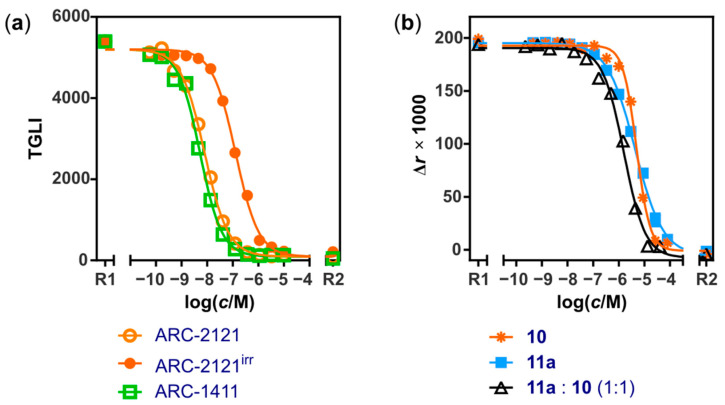
Displacement assays of the photocleavable ARC-2121 and reference compounds. (**a**) Displacement of photoluminescent probe ARC-1182 (10 nM) from the complex with PKAcα (1 nM) by ARC-1411 (green rectangles) and ARC-2121 (orange spheres) before (open symbols) and after (filled symbols) irradiation. The TGLI signal corresponding to the ARC-1182:PKAcα complex was measured. (**b**) Displacement of photoluminescent probe ARC-583 (2 nM) from the complex with PKAcα (3 nM) by **11a** (blue rectangles), **10** (orange asterisks) and 1:1 mixture of **11a** and **10** (black triangles, the concentration corresponds to that of a single component). (**a**,**b**) Reference solutions contained the corresponding photoluminescent probes in the presence (R1) or in the absence (R2) of PKAcα. 3.

Next, the photocleavable inhibitor ARC-2121 was characterized by the competitive equilibrium binding assay. The measured *K*_D_ value for ARC-2121 (19 ± 7 pM; Table 1; Figure 6a) was around six times higher than the *K*_D_ value of ARC-1411, showing that the incorporation of the bulky photocleavable NDBF-β-amino acid into the structure somewhat reduced the affinity. However, such modification was still well tolerated by PKAcα. This result is in agreement with observations of the previous structure-activity study of ARC-type inhibitors of basophilic PKs [18]. ARC-2121 binds to PKAcα as strongly as the high-affinity photoluminescent probe ARC-1182.

If compared to the millimolar inhibitory potency of hexa-d-arginine [34], the reference compound **10** possessed much higher affinity (Table 1; Figure 6b), which suggests that the NDBF group may weakly interact with the ATP-binding cavity of PKAcα and **10** is a very weak inhibitor of PK. The affinities of **11a** and **10** were still considered sufficiently low as the corresponding *K*_D_ values were in sub-micromolar region, close to the *K*_D_ value of **2a**.

The Irradiation of the assay mixtures in 384-well microplate was performed with a custom-made device with an array of 365 nm LED-s fixed at distances corresponding to this microplate format (Figures S2 and S3). It was previously shown that the used irradiation conditions did not interfere with the activity of PKAcα or the output signal of the assay [22]. In sharp contrast to the results obtained with the redox-sensitive ARC-2194, the 2-min treatment of ARC-2121 with UV-radiation at 365 nm did not result in a dramatic drop of the affinity. Irradiated ARC-2121 still possessed very high apparent affinity toward PKAcα, as shown by the 30-fold higher apparent *K*_D_^app^ value (0.6 ± 0.3 nM, Table 1; Figure 6a). Theoretically, if ARC-2121 had been quantitatively photolyzed into the 1:1 mixture of **11a** and **11b** (Scheme 3), the apparent affinity of the irradiated inhibitor would have been at least 10,000-fold lower, close to the sub-micromolar *K*_D_^app^ value for 1:1 mixture of **11a** and **10** (Table 1; Figure 6b). The displacement curves were identical after 2 min irradiation. HPLC-MS monitoring of the photochemical reaction did not detect ARC-2121 even after 15 s of irradiation, and no significant changes in the chromatogram were observed after 30 s of irradiation. However, the photolysis of ARC-2121 turned out to yield a complex mixture of products from which only **11a** could be identified by HPLC-MS (Figure 4, Figures S4 and S5). Based on these data, it could be speculated that the high residual apparent affinity upon irradiation of ARC-2121 could result from the combination of incomplete photolysis and the formation of excessively active products.

## 3. Discussion

Photo-responsive inhibitors have been developed for controlling the activity of a variety of PKs, e.g., PKAcα [22,45,46], MLCK [45], Src [29], MAPK [47] and CK1 [47], to name a few (for a recent review, see [2]). These inhibitors include photocaged, photoswitchable, and photo-deactivatable variants and the active forms of the inhibitors are mostly either targeting the ATP-binding site or protein-substrate binding site of the PKs. One example describes the photo-deactivatable biligand inhibitor of Src, which simultaneously associates with the SH1 and SH2 domains of the PK [29].

In our recent report we demonstrated that bisubstrate inhibitors of basophilic PKAcα can be efficiently photocaged by attachment of a single NDBF photocage to a hot-spot position on the ATP-binding site-targeting fragment [22]. The present work demonstrates that deactivatable bisubstrate inhibitors of PKs can be developed by incorporating a cleavable junction into the linker region. We focused on basophilic PK inhibitors and used two different stimuli-responsive junctions, one cleavable by irradiation at 365 nm and the other by chemical reduction, to modulate the affinity of bisubstrate inhibitors. 

In general, deactivation of an inhibitor can be observed by a shift of a dose-response curve in a biochemical assay, which is caused by the increase of IC_50_ value from IC_50_^ON^ (the IC_50_ of the active inhibitor) to IC_50_^OFF^ (the IC_50_ of the deactivated inhibitor). The fraction of the active enzyme before and after applying the trigger depends on the initial concentration of the deactivatable inhibitor. If the sigmoidal dose–response model describes the concentration dependencies of the inhibition before and after applying the stimulus, the biggest change of the fraction of the active enzyme in the solution is observed at the concentration of the inhibitor *I*_opt_ whose logarithm is the average of the corresponding logIC_50_ values (Equation (2)):

(2) $$\log I_{\mathrm{opt}}=\frac{1}{2}\left(log{\mathrm{IC}}_{50}^{\mathrm{ON}}+log{\mathrm{IC}}_{50}^{\mathrm{OFF}}\right)$$

The ratio of fractions of active enzyme after (*a*^max^_opt_) and before (*a*^min^_opt_) deactivation of the inhibitor (i.e., the on/off ratio of the activity of the enzyme) is determined by the square root of the ratio of the corresponding IC_50_ values (Equation (3)):

(3) $$\frac{a_{\mathrm{opt}}^{\max}}{a_{\mathrm{opt}}^{\min}}={\left(\frac{{\mathrm{IC}}_{50}^{\mathrm{OFF}}}{{\mathrm{IC}}_{50}^{\mathrm{ON}}}\right)}^{1/2}$$

**Figure 7:**
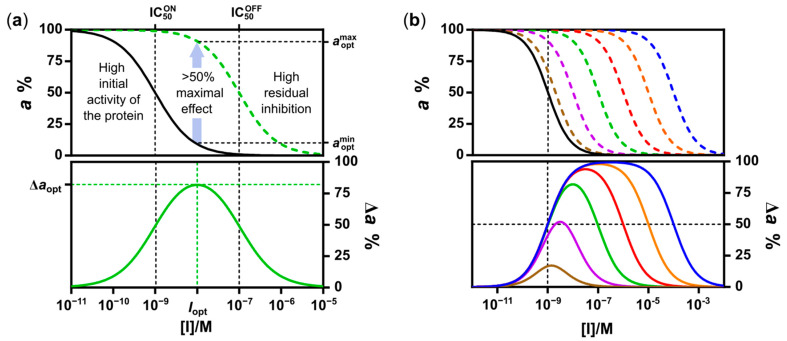
Semi-logarithmic plots of simulated curves (1:1 reversible binding model without ligand depletion) explaining the theoretical gain of activity of an enzyme upon deactivation of an inhibitor. IC_50_^ON^ and IC_50_^OFF^ are the IC_50_ values of the inhibitor pre- (solid black curve IC_50_^ON^ = 10^−9^ M) and post-deactivation (dashed curves), and *a* is fraction of the active enzyme. The bottom panels show difference of the curves obtained post- and pre-deactivation. (**a**) green curves correspond to IC_50_^OFF^ = 10^−7^ M; (**b**) the dashed curves correspond to IC_50_^OFF^ = {2 × 10^−9^; 10^−8^; 10^−7^; 10^−6^; 10^−5^ and 10^−4^ M} from left to right, respectively. The same color-coding is used in the bottom panel.

Thus, the larger the difference between IC_50_^ON^ and IC_50_^OFF^ values is (ΔIC_50_), the higher the dynamic range of activity change (Δ*a*_opt_ = *a*^max^_opt_ − *a*^min^_opt_) of the enzyme is. Within the concentration range between IC_50_^ON^ and IC_50_^OFF^, activity change of the enzyme is higher than 50% of Δ*a*_opt_ (Figure 7a). If the concentration of the deactivatable inhibitor is lower than IC_50_^ON^, basal activity of the enzyme is high in the presence of the intact deactivatable inhibitor. If the concentration of the deactivatable inhibitor is higher than IC_50_^OFF^, the post-deactivation residual inhibition is high, and thus no significant activation of the enzyme occurs.

Small ΔIC_50_ induces a small dynamic range of activity change of the enzyme with an optimum within a narrow concentration range of the inhibitor. Large ΔIC_50_ leads to a high dynamic range within a wide concentration range of the inhibitor (Figure 7b). In case of inhibitor depletion (i.e., if the inhibitor is tight-binding before deactivation), IC_50_^ON^ increases and the ΔIC_50_ becomes smaller than it would be in conditions where the IC_50_-*K*_D_ relationship is linear (i.e., in the absence of tight-binding conditions). At the same time, the maximal activity change of the enzyme could occur at concentration of a tight-binding inhibitor that is close to concentration of the enzyme. The preceding discussion also applies in the case of activatable (e.g., photocaged) inhibitors, except that transition of affinity of the inhibitor (and, correspondingly, concentration change of the active enzyme) occurs in the opposite direction.

With the redox-deactivatable inhibitor, ARC-2194, an over four orders of magnitude increase of *K*_D_^app^ and inhibition IC_50_ was established, which corresponds to Δ*a*_opt_ of 98% (on/off ratio of the enzyme activity is more than 100). Irradiation of the photo-deactivatable inhibitor ARC-2121 at 365 nm increased the *K*_D_^app^ about 30-fold, that would lead to Δ*a*_opt_ of 70% (5.6-fold increase of the enzyme activity). The good correlation between the *K*_D_ values established by equilibrium binding assay and IC_50_ values obtained by enzyme kinetic assay (Table 1) supports the use of the *K*_D_ differences of the compounds to estimate the performance of the compounds in functional assays.

The highest ΔIC_50_ value for a deactivatable bisubstrate inhibitor with a cleavable linker is obtained if: (1) incorporation of a cleavable moiety into the linker region is well tolerated by the target enzyme (i.e., the affinity of the intact inhibitor is high), (2) produced fragments of the inhibitor possess low affinity, and (3) the cleavage reaction proceeds with a high yield [48]. The last two factors, the affinity of the most potent fragment and yield of the transformation, determine the IC_50_^OFF^ value of a deactivatable inhibitor. In the case of two bisubstrate inhibitors with the same affinity, a higher ΔIC_50_ is expected with the compound whose fragments have an equal contribution on the binding energy, unless the change of functional groups during fragmentation strongly contributes on the change of affinity.

The *K*_D_ values of the novel inhibitors, ARC-2121 and ARC-2194, were in one- and two-digit picomolar range, close to the corresponding value for the non-cleavable lead compound ARC-1411. The insertion of the cleavable junction into the structure of ARC-1411 in both cases decreased the affinity of binding to PKAcα. However, this decrease was still marginal, which demonstrates that the cleavable linkers in these positions were well tolerated by PKAcα. The ATP-binding site-targeted moiety of an ARC-type bisubstrate inhibitor has higher contribution to the binding energy of the inhibitor than its peptide fragment. This is revealed by the equality of *K*_D_ value of **2a** and the *K*_D_^app^ value of the disassembled ARC-2194. A lower-affinity fragment in the position of ATP-binding site-targeting moiety would have increased both IC_50_^ON^ and IC_50_^OFF^ values. However, in the present study, the choice of 7DP-Pip-residue in the role of ATP-site targeting fragment enabled the exact calculation of ΔIC_50_ (or Δ*K*_D_) upon cleavage of the linker, because the relatively high (submicromolar) affinity of **2a** was in the measurement range of the used assay systems.

The yield of transformation of an inhibitor into the low-affinity form considerably contributes to ΔIC_50_. Efficient deactivation of an inhibitor relies on close to quantitative chemical (or photochemical) transformation. This is in contrast with photocaged inhibitors, which can be sufficiently activated already after incomplete conversion. For example, if the deactivated form of an inhibitor possesses no measurable affinity and the conversion proceeds with a 50% yield, the apparent IC_50_ value is only twice as high as after quantitative transformation. The relative change of IC_50_ would be very large in the case of a photocaged inhibitor (from infinity to 2 × IC_50_ of the active inhibitor), but very small (twofold) in the case of a deactivatable inhibitor. Accordingly, a 99% yield of the transformation of the deactivatable inhibitor would cause a hundredfold increase of the IC_50_ value, which is remarkable, but still much less than it would be observed after 100% conversion into a completely inactive form. In other words, incomplete deactivation of a deactivatable inhibitor and premature activation of a photocaged inhibitor substantially reduce the dynamic range of the activity change of the target enzyme. At the same time, a minor contamination with the transformation product of the batch of the inhibitor (e.g., arising from premature activation/inactivation) considerably decreases the IC_50_ change of an activatable inhibitor, but leads to a marginal effect on the IC_50_ change of a deactivatable inhibitor. The latter point makes the preparation and handling of deactivatable inhibitors more convenient. However, if compared to activatable inhibitors, deactivatable inhibitors are less robust as they require thorough optimization of reaction conditions (e.g., dose of irradiation, reaction time, concentration of the inhibitor) if the highest dynamic range of protein activation is sought.

The reductive cleavage of ARC-2194 proceeded quantitatively upon the addition of reducing agent TCEP in excess. This was demonstrated by HPLC-MS monitoring of the process (Figure 4a) and the *K*_D_^app^ value obtained after TCEP-treatment, which coincided with the *K*_D_ of **2a** (Figure 3). The reasons why the apparent affinity change of photo-deactivatable ARC-2121 was much smaller compared to ARC-2194 remained unclear. The HPLC monitoring of the cleavage mixture shows that the photolysis reaction is much more complex than it is described by the route presented in Scheme 3. Possibly, the main contribution on the affinity observed upon irradiation still originated from the presence of a small amount of unreacted ARC-2121 in the cleavage mixture that was undetectable by MS. Indeed, if the cleavage of the inhibitor had yielded completely inactive fragments, a 30-fold decrease of apparent affinity would only correspond to about 3% of the residual ARC-2121 in the solution. However, the possibility of formation of bisubstrate PK inhibitor-like structures during the photolysis of ARC-2121 cannot be excluded. It is likely that replacing NDBF-β-amino acid residue with a photocleavable self-immolative ‘traceless’ linker could decrease the probability of formation of structures of the latter type. The ATP-binding site of PKs binds flat aromatic moieties. If compared to the low affinity of hexa-d-arginine, the relatively high affinity of **10** (as well as the photocaged bisubstrate inhibitor ARC-2113 [22]) suggests that the photo-responsive NDBF moiety itself or its derivatives are good candidates for the role of the ATP-binding site-targeting fragments of photo-deactivatable bisubstrate PK inhibitors (according to the principle presented in Figure 1c). However, the statistically significant 30-fold affinity decrease of ARC-2121 upon photolytic cleavage is comparable to switching factors reported for efficiently photoswitchable compounds [47].

Compounds with switchable bioactivity offer an alternative to irreversibly deactivatable ligands. They have a clear advantage of being ‘regenerated’ upon deactivation, differently from cleavable compounds, whose regeneration requires chemical recombination of the fragments. However, irreversibly deactivatable ligands may have advantages for some developments. First, the photocleavage-based irreversible transition typically results in greater differential activity between the high- and low-affinity form of the ligand than it is achievable with photoswitchable derivatives [47,49]. Second, the photoswitchable compounds are prone to relax spontaneously to the isomer with opposite configuration when illumination is stopped. Third, the structural variability of known photoswitchable moieties is much smaller if compared to the photocleavable structures that limits opportunities to design ligands and to avoid side reactions associated with particular structures. For example, widely used photoswitchable moieties are diarylazo-compounds, which are prone to undergo glutathione-mediated reduction to the corresponding hydrazines or decomposition by other metabolic processes which reduces their lifespan in the contact with biological environment [47,50]. Finally, the combination of spatiotemporal activation and permanent deactivation enables to focus the activity of a drug or an agrichemical only to the site of action, which is highly desirable for reducing systemic and environmental toxicity of these types of bioactive compounds [25,49,51]. Shortening the duration of action for escaping side effects is the goal of the development of photopharmaceuticals [25] and self-destruct drugs. Importantly, as suggested by the results of this and a previous report [22], the implementation of irreversible temporary activation is very straightforward in the case of bisubstrate inhibitors of PKs (and bivalent ligands in general) if two semiorthogonal responsive moieties are incorporated into the structure whose sequential cleavage first activates and then deactivates the inhibitor (Figure 1f).

In contrast to the conventional inhibitors, deactivatable inhibitors (and deactivatable ligands in general) possess two opposite features, namely the ability to efficiently capture and release active enzymes. Therefore, these compounds could be of the highest value in applications where temporal capture of the target enzyme is desired, such as affinity matrices for fishing the proteins out of the solution and temporal immobilization of the active forms of the enzymes. In addition, deactivatable inhibitors could be used for the development of reagents for site-specific affinity-labeling of enzymes [52]. Deactivatable inhibitors could also be used as selective enzyme-stabilizing/catalytic site-protecting agents, which in response to the trigger would allow restoring enzyme activity without the requirement for additional separation steps. Similarly, deactivatable inhibitors could be used to block the enzymatic activity until sudden activation is required, e.g., for starting the enzyme-catalyzed reaction at a fixed time point in kinetic experiments. In these applications, both light- and redox-responsive inhibitors could be valuable.

The development of bivalent ligands is generally justified by the aim to increase the selectivity and affinity of the interaction, or to specifically target dimeric complexes. The present report highlights yet another aspect, the modularity of bivalent ligand design, which provides unique advantages for the development of stimuli-responsive bioactive compounds.

## 4. Materials and Methods

### 4.1. Equipment and Software

^1^H NMR (700 MHz) and ^13^C NMR (176 MHz) spectra were acquired with Bruker Avance-III 700 MHz NMR spectrometer (magnetic field 16.4 T). Tetramethylsilane (TMS) was used as the internal standard.

HPLC analysis and purification of novel compounds was performed using a Shimadzu Prominence LC Solution HPLC system with SPD M20A PDA and ESI-MS LCMS-2020 detectors. Purification was performed using the reverse phase column Luna C18 (250 × 4.6 mm, particle size 5 μm) eluted with MeCN/H_2_O gradient (0.1% TFA) at flow rate of 1 mL/min. The column was thermostated at 40 °C. The purification of light-sensitive ARC inhibitors was carried out with a switched-off UV-Vis detector and the fraction was collected based on previously established *R*_t_ of the target compound.

High resolution mass spectra (HRMS) were obtained on Thermo Electron LTQ Orbitrap (ESI-HRMS) in positive ion mode or with combined Varian 910-FT-ICR and Varian J-320 3Q spectrometers in positive ion mode.

All procedures with photocleavable substances were performed under red light (Paulmann RGB LED GSL, 5W, peak emission at 630 nm) to avoid premature photolysis of the compounds. Photolysis was performed with timer-equipped custom-made LED array accommodating 16 LEDs [150 mcd, *λ*_max_ = 365 nm (Figure S3), 975 mW, part number LTPL-C034UVH385] designed for irradiation of one row of wells at a time in a 384-well microtiter plate (Figure S2). Photolysis experiments were performed in black 384-well polystyrene microplates at 5 mm distance between the sample and the light source. The photolysis was carried out at ambient temperature.

UV-Vis spectra of ARC inhibitors were obtained with NanoDrop 2000c spectrometer (Thermo Scientific). Concentrations were calculated according to the Beer–Lambert law using the following molar extinction coefficients: 250,000 M^−1^cm^−1^ for ARC-1182 and ARC-1063 at 652 nm (λ_max_ of the respective fluorescent dyes PromoFlour-647 and AlexaFluor-647); 80,000 M^−1^cm^−1^ for ARC-583 at 558 nm (λ_max_ of TAMRA fluorescence dye); 18,400 M^−1^cm^−1^ for **10** and ARC-2121 at 340 nm; 16,000 M^−1^cm^−1^ for **2a, 11a**, ARC-2194, and ARC-1411 at 287 nm; 8247 M^−1^cm^−1^ for the peptide substrate of PKAcα (Cysteine Sox Kinase Sensor, code AQT0458, in 0.1 M NaOH + 1 mM EDTA) at 360 nm, and 4400 M^−1^cm^−1^ for H-89 at 323 nm.

Buffered aqueous solutions of biologically active compounds were prepared in low-binding centrifuge tubes (Protein LoBind, Eppendorf AG), except photocleavable substances, which were stored in amber tubes (Axygen #MCT-150-X). The biochemical binding assays and measurements of the catalytic acitivity of PKAcα were performed on black non-binding-surface 384-well polystyrene microplates (Corning #4514).

Eppendorf Research (Eppendorf) pipettes with PP pipet tips (low-retention RPT, Starlab GmbH, or Nerbe Plus premium surface) and 12-channel electronic pipettes with original tips (E1 ClipTip, Thermo Scientific) were used for liquid handling in biochemical assays.

The fluorescence intensity (FI), fluorescence anisotropy (FA), and time gated photoluminescence intensity (TGLI) measurements were performed with a PHERAstar (BMG Labtech) microplate reader. The FI and FA measurements of the photoluminescent probe ARC-583 were performed with FP optical module [EX 540(50) nm, EM 590(50) nm]. The detector was adjusted with solution of the free probe ARC-583 (in the absence of PKAcα). The FI measurements of the peptide substrate of PKAcα were performed with FI optical module (EX 370(10) nm, EM 530(10) nm). The TGLI measurements of photoluminescent probes ARC-1182 and ARC-1063 were performed with HTRF optical module (EX 330(50) nm, EM 675(50) nm). The TGLI was recorded for 200 µs after 60 µs delay from the flash-excitation.

Biosan CH-100 heating/cooling dry block was used for thermostating the reaction between ARC-2194 and TCEP that was monitored by RP HPLC.

Data were processed with Graphpad Prism software (v 5.00 and 6.04, GraphPad Software, La Jolla, CA, USA) and Microsoft Excel (v 2203, Redmond, WA, USA).

### 4.2. Reagents

Chemicals from the following commercial sources were used: Acros, Alfa Aesar, Bachem, Caslo ApS (Fmoc-[d-Arg(Pbf)]_6_-Rink amide resin (0.18 mmol/g)), Calbiochem, Cayman Chemical (H-89 hydrochloride), Deutero GmbH (deuterated solvents), Fischer Scientific, Fluka, Fluorochem, Iris Biotech GmbH (reagents for Fmoc-peptide synthesis), Lachner, Macherey-Nagel (TLC plates), Mallinckrodt, Merck, Reachim, Riedel-de Haën, Santa Cruz and Sigma Aldrich. The peptide substrate of PKAc (Cysteine Sox Kinase Sensor code AQT0458) was obtained from AssayQuant Technologies Inc. Human recombinant full-length PKAc α-isoform was obtained from Biaffin. Upon arrival, the stock solution of PKAcα was divided into batches and stored at −80 °C. After removal from a freezer at −80 °C, the batches of PKAcα were stored at 0 °C in an ice box until consumed, to avoid repetitive freeze-thaw cycles.

Dess Martin Periodinane (DMP), ARC-1411 [31], ARC-1182 [39], ARC-583 [40], ARC-1063 [38], 4-(piperazin-1-yl)-7H-pyrrolo [2,3-d]pyrimidine (7DP-Pip, **2a**) [24], and 3-nitro-4-bromodibenzo[*b*,*d*]furan [22] had been previously synthesized in the same laboratory.

THF was distilled from sodium benzophenone ketyl. Other chemicals were used as provided by the manufacturer.

### 4.3. HPLC-MS Analysis of the Fragmentation of ARC-2121 and ARC-2194 

The reaction mixture containing ARC-2194 (100 µM) and TCEP (1 mM) in buffer (50 mM HEPES, 150 mM NaCl, 0.005% Tween-20, pH = 7.5) was incubated at 30 °C. At fixed time points (1, 10, 30, 60, and 120 min), 10 µL of the solution (corresponding to 1 nmol of the substrate) was injected into the RP-HPLC column. The column was eluted with 0.1% TFA/(5–35% MeCN/H_2_O in 12 min) at flow rate of 1 mL/min.

ARC-2121 (200 µM) was dissolved in H_2_O:MeCN (10:1). ARC-2121 (200 µM, 20 µL) was irradiated for a fixed time (15, 30, 60, 90, and 120 s) inside a microtiter plate at room temperature (rt) using the custom-made 365 nm LED array (Figure S2). Then, 9 µL (1.7 nmol) of the solution was injected into the RP-HPLC column. The column was eluted with 0.1% TFA/(5–35% MeCN/H_2_O in 12 min) at a flow rate of 1 mL/min (Figures S4 and S5).

### 4.4. Biochemical Binding/Displacement Assay

The biochemical binding/displacement assays were performed in a final volume of 20 μL per well of the microtiter plate, in a buffer solution containing 50 mM HEPES (pH = 7.5), 150 mM NaCl, 0.005% Tween-20 (P20) and 5 mM DTT, except in experiments presented in Figure 3 where DTT was excluded to avoid premature cleavage of ARC-2194. The microplates were incubated for 30 min at 30 °C before measurements. The measurements were repeated after 60 min to verify that the reactions had reached the equilibrium. The concentration of the active form of PKAcα was determined on each day of experiment prior to performing the displacement assay by titration of a fixed concentration of photoluminescent probe ARC-1063 (10 nM; *K*_D_*= 0.015 nM) [39], ARC-1182 (10 nM; *K*_D_* = 0.015 nM) [39], or ARC-583 (20 nM; *K*_D_* = 0.5 nM) [40] with 2-fold dilutions of the enzyme. Formation of the complex was observed by the change of FA and the concentration of PKAcα was calculated as previously described [40,42]. The equilibrium dissociation constants ($K_{D}$) of the complexes of characterized inhibitors with PKAcα were established using FA-based (2 nM ARC-583 with 3 nM PKAcα) [39,40] and TGLI-based (10 nM ARC-1063 or ARC-1182 with 1 nM PKAcα) [31,38] competitive binding/displacement assays, following the corresponding previously published procedures. The apparent affinity (*K*_D_^app^) of photolyzed ARC-2121 was determined after irradiation of the solutions under 365 nm LED for 2 min. The experiments presented in Figure 3 were performed as follows. First, the solutions were incubated for 30 min at 30 °C, thereafter the FA or TGLI signals were measured. Then, the stock solution of TCEP (1 µL) was added to all wells to the final concentration of 1 mM. The microplates were then incubated for an additional 30 min at 30 °C and the FA or TGLI signals were measured in the presence of TCEP. All experiments were carried out in at least two replicates (*n* ≥ 2) and the reported errors are the standard errors of the mean.

The anisotropy change (Δ*r*) with respect to the anisotropy of free photoluminescent probe ARC-583 and the TGLI of the photoluminescent probes ARC-1182 and ARC-1063 were plotted as functions of log[I]_T_, where [I]_T_ corresponds to the total concentration of the ‘dark’ ligand/inhibitor that competes with the probe for the binding to the catalytic subunit of PK ([I]_T_ = [I] + [PK:I], where [I] and [PK:I] correspond to the equilibrium concentrations of unbound I and PK:I complex, respectively). The *K*_D_ values of the characterized inhibitors were calculated by fitting the data to the previously published exact one-site competitive binding model explained in Appendix A [40,42,53].

### 4.5. Kinase Activity Assay

The catalytic activity of PKAcα was measured by the PhosphoSens^®^ technology (AssayQuant Technologies Inc) according to the manufacturers’ recommendations. The phosphorylation mixtures contained the inhibitors (ARC-2194 or H89; 3-fold dilutions) PKAcα (0.5 nM active catalytic subunit), ATP (1 mM), Mg(OAc)_2_ (10 mM), TCEP (0 or 1 mM), peptide substrate Cysteine Sox Kinase Sensor (10 µM), HEPES (50 mM, pH = 7.5), NaCl (150 mM), P20 (0.005%), and Na_3_EDTA (1 mM). The solutions were pre-incubated for 0.5 h at 30 °C before starting the phosphorylation by the addition of the peptide substrate. The progress of the phosphorylation at 30 °C was monitored by the change of fluorescence intensity by PHERAStar microplate reader in 90 s intervals. The data were corrected for lag times and the slopes of fluorescence intensity vs. time dependencies were calculated using the linear region of the data (the first 20 min). The reference solutions without the inhibitors and without PKAcα were used as positive and negative controls of the catalytic activity of the enzyme, respectively.

### 4.6. Chemical Synthesis

#### 4.6.1. Solid Phase Synthesis

The novel ARC inhibitors **10**, **11a**, ARC-2121, and ARC-2194 were synthesised in 3.3…14 µmol scale, following the standard Fmoc-solid phase peptide synthesis protocols starting from Fmoc-Rink Amide MBHA resin (0.61 mmol/g) for **11a** or Fmoc-[d-Arg(Pbf)]_6_-Rink amide resin (0.18 mmol/g) for other compounds. The solid phase synthesis was performed in polyproylene (PP) SPE tubes equipped with PP filters (70-μm porocity). Procedures involving light-sensitive compounds were carried out under red light. The following building blocks were used: 4-(piperazin-1-yl)-7*H*-pyrrolo [2,3-d]pyrimidine (7DP-Pip, **2a**, CAS 252722-52-4), nonanedioic acid (Nda), dibenzofuran derivatives **4** and **8**, and *C*,*C*′-(dithiodi-2,1-ethanediyl)dbis[*C*-(2,5-dioxo-1-pyrrolidinyl) carbonate] (CAS 1688598-83-5, NHS-activated disulfide linker). Nda and 7DP-Pip were incorporated into the structures as described by Ivan et al. [31]. Disulfide linker was attached to the structures by the following protocol. First, amino-functional resin was treated with NHS-activated disulfide linker (9 eq) and TEA (4 eq) in DMF (2.5 h, rt). Then the resin was washed with DMF. Second, a suspension of **2a** (3 eq) in the solution of TEA (4 eq) in DMF was added and the mixture was shaken overnight. The hydroxyl group of compound **9** (15 μmol, 1 eq) was oxidized to carbonyl by treatment with DMP (19.15 mg, 45.15 μmol, 3 eq) inside SPPS vessel in DMF (1 mL) for 3.5 h at rt. Finally, the resins were washed with DMF (3 × 1 min), iPrOH (1 min), and DCM (3 × 1 min) and dried in vacuum. The target compounds were cleaved from the resin using H_2_O:TFA:TES (95:2.5:2.5, v:v:v) coctail (3 h, rt) and purified by RP-HPLC. UV-Vis spectroscopy, ESI MS and ESI HRMS were used for structure verification. The HPLC gradients, retention times, peak area % of the purified compounds, and MS data are given in Table S1. The HPLC chromatograms and the corresponding UV-Vis spectra of the peaks of the compounds are given in the Supplementary Material.

#### 4.6.2. Compound **5**

2-Bromomethyl-3-nitrodibenzo[*b*,*d*]furan [22] (70.7 mg, 0.23 mmol, 1 eq) was added to the solution of *N*-methylmorpholine *N*-oxide (85 mg, 0.73 mmol, 3.2 eq) in dry THF (2 mL) at rt. [37] The solution was refluxed overnight and cooled to rt. The reaction mixture was analyzed using TLC (100% CHCl_3_, *R*_f_ = 0.4). Water (2 mL) was added and the mixture was extracted with Et_2_O (3 × 1 mL). The organic layer was washed with brine, dried over Na_2_SO_4_, concentrated, and purified by using normal phase column chromatography. The product (55.7 mg, 63% yield) was a faint-yellow solid, analyzed using NMR. **NMR** **δ_1H_**(700 MHz; CDCl_3_; Me_4_Si) 10.50 (1 H, s, C**H**O); 8.56 (1 H, s, **H**_ar_); 8.32 (1 H, s, **H**_ar_); 8.08 (1 H, d, *J* = 7.7, **H**_ar_); 7.69 (1 H, d, *J* = 8.4, **H**_ar_); 7.66–7.64 (1 H, m, **H**_ar_); 7.51–7.49 (1 H, m, **H**_ar_). **NMR** **δ_13C_**(176 MHz; CDCl_3_; Me_4_Si) 187.74, 158.65, 157.06, 130.29, 129.36, 127.23, 124.59, 122.20, 122.13, 122.09, 112.54, 108.88.

#### 4.6.3. Compound **6**

2-formyl-3-nitrodibenzo[*b*,*d*]furan (compound **5**; 24.2 mg, 0.1 mmol, 1.1 eq) and Ti(OEt)_4_ (43.8 mg, 0.18 mmol, 2 eq, 0.5 M) were dissolved in THF (365 μL) under N_2_ atmosphere. (*R*)-*tert*-Butanesulfineamide (11.5 mg, 0.1, 1 eq) was added to the solution and stirred at rt for 7 h. The reaction mixture was diluted with EtOAc, washed with brine, dried over Na_2_SO_4_, concentrated, and purified using normal phase column chromatography. The product (24.1 mg; 70% yield) was analyzed using NMR. **NMR** **δ_1H_**(700 MHz; CDCl_3_; Me_4_Si) 9.09 (1 H, s, **H**C = N); 8.54 (1 H, s, **H**_ar_); 8.25 (1 H, s, **H**_ar_); 8.08 (1 H, d, *J* = 7.7, **H**_ar_); 7.67 (1 H, d, *J* = 8.4, **H**_ar_); 7.64–7.62 (1 H, m, **H**_ar_); 7.49–7.47 (1 H, m, **H**_ar_); 1.35 (9H, s, C(C**H**_3_)_3_). **NMR** **δ_13C_**(176 MHz; CDCl_3_; Me_4_Si) 159.78, 158.51, 156.24, 147.71, 130.03, 129.03, 124.75, 124.29, 122.20, 122.17, 122.16, 121.98, 112.46, 108.87, 58.39, 22.75.

#### 4.6.4. Compound **7**

LiCl (45.0 mg, 1 mmol, 15 eq) and Zn powder (93.8 mg, 1.4 mmol, 20 eq) were mixed and dried in a flame-dried flask and heated at 200 °C for 20 min under vacuum [36] Solution of Me_3_SiCl (1.3 μL, 0.01 mmol, 0.15 eq) and 1,2-dibromoethane (4.5 μL, 0.05 mmol, 0.75 eq) in dry THF were added to the mixture. Next, *tert*-butyl bromoacetate (260 μL, 1.7 mmol, 25 eq; 2.7 M in THF) was added at rt. After stirring for 1 h, the solution was transferred to a flame-dried flask with compound **6** in THF (1.2 mL) at 0 °C and stirred for 4 h. The reaction mixture was diluted with EtOAc, washed with citric acid (0.25 M), NaHCO_3_ (5%), and brine, dried over Na_2_SO_4_, concentrated, and purified using normal phase column chromatography. The product (22.8 mg; 71% yield) was analyzed with NMR. **NMR** **δ_1H_**(700 MHz; CDCl_3_; Me_4_Si) 8.22 (1 H, s, **H**_ar_); 8.18 (1 H, d, *J* = 0.7, **H**_ar_); 7.99 (1 H, d, *J* = 7.7, **H**_ar_); 7.64 (1 H, d, *J* = 7.7, **H**_ar_); 7.60–7.58 (1 H, m, **H**_ar_); 7.45–7.43 (1 H, m, **H**_ar_); 5.53–5.50 (1 H, m, N-C**H**); 5.00 (1 H, d, *J* = 4.9, N**H**); 3.16 (1 H, dd, *J* = 4.9, *J* = 16.1, **H**CH); 3.01 (1 H, dd, *J* = 7.0, *J* = 16.1, HC**H**); 1.37 (9 H, s, (C**H**_3_)_3_); 1.24 (9 H, s, (C**H**_3_)_3_).

#### 4.6.5. Compound **3**

Compound **7** (22.8 mg, 0.05 mmol) was treated with a mixture of 1 M HCl (2 eq) in Et_2_O and EtOH (1.1 eq). The reaction mixture was stirred at rt for 35 min, concentrated in vacuo and triturated with Et_2_O. The product was dissolved in DCM:MeOH (1:1, v:v) and stirred at 0 °C for 30 min. Fmoc *N*-hydroxysuccinimide ester (26.7 mg, 0.075 mmol, 1.5 eq) was added to the mixture, followed by triethylamine (TEA, 20 μL, 0.15 mmol, 3 eq). The reaction mixture was stirred overnight at rt, concentrated in vacuo, resuspended in ethyl acetate and washed with water. The organic phase was dried over Na_2_SO_4_, concentrated in vacuo, and purified with normal phase column chromatography. As the *tert*-butyl ester had not been cleaved with the HCl treatment (confirmed by NMR and HPLC-MS analysis), the *tert*-butyl ester was cleaved using TFA:H_2_O:TES mixture (90:5:5, v:v:v; 555 μL) stirring for 3 h at rt. The mixture was concentrated in vacuo resulting in 13 mg of product (50% yield over 3 steps). **NMR** **δ_1H_**(700 MHz; DMSO; Me_4_Si) 8.42 (1 H, s, **H**_ar_); 8.34 (1 H, s, **H**_ar_); 8.26 (1 H, d, *J* = 7.7, **H**_ar_); 8.14 (1 H, d, *J* = 7.7, **H**_ar_); 7.82–7.79 (2 H, 2×m, **H**_ar_); 7.67–7.65 (1 H, m, **H**_ar_); 7.62 (1 H, d, *J* = 7.7, **H**_ar_); 7.60 (1 H, d, *J* = 7.7, **H**_ar_); 7.53–7.51 (1 H, m, **H**_ar_); 7.34–7.30 (2 H, 2×m, **H**_ar_); 7.24–7.22 (1 H, m, **H**_ar_); 7.20–7.17 (1 H, m, **H**_ar_); 5.50–5.47 (1 H, m, N-C**H**); 4.23–4.21 (2 H, m, O-C**H_2_**); 4.16 (1 H, m, CH_2_-C**H**); 2.86 (1 H, dd, *J* = 9.8, *J* = 16.1, **H**CH); 2.79 (1 H, dd, *J* = 4.2, *J* = 16.1, HC**H**).

#### 4.6.6. Compound **8**

LiCl (72.4 mg, 1.66 mmol, 1.5 eq) and Zn powder (154.7, 2.3 mmol, 2.1 eq) were mixed and dried in a flame-dried flask at 200 °C for 20 min under vacuum. Solution of Me_3_SiCl (2.1 μL, 0.02 mmol, 0.02 eq) and 1,2-dibromoethane (7.2 μL, 0.05 mmol, 0.08 eq) in dry THF were added to the mixture. Next, *tert*-butyl bromoacetate (250 μL, 1.7 mmol, 18 eq) was added at rt. After stirring for 1 h, the solution was transferred to a flame-dried flask with compound **5** in THF (2 mL) at 0 °C and stirred overnight. The reaction mixture was diluted with EtOAc, washed with aqueous HCl (1 M), and extracted with EtOAc (3x). The organic layers were combined, dried over MgSO_4_, concentrated, and purified with normal phase column chromatography. The product (31,9 mg; 98% yield) was analyzed with NMR. **NMR** **δ_1H_**(700 MHz; CDCl_3_; Me_4_Si) 8.46 (1 H, s, **H**_ar_); 8.21 (1 H, s, **H**_ar_); 8.02 (1 H, d, *J* = 7.7 Hz, **H**_ar_); 7.63 (1 H, d, *J* = 8.4 Hz, **H**_ar_); 7.60–7.57 (1 H, m, **H**_ar_); 7.44–7.42 (1 H, m, **H**_ar_); 5.78 (1 H, dt, *J* = 9.1, *J* = 2.8 Hz, C**H**-OH); 4.15 (1 H, d, *J* = 2.8 Hz, O**H**); 2.98 (1 H, dd, *J* = 16.8, *J* = 2.8 Hz, **H**CH-COO); 2.65 (1 H, dd, *J* = 16.8, *J* = 9.1 Hz, HC**H**-COO); 1.49 (9 H, s, (C**H**_3_)_3_). **NMR** **δ_13C_**(176 MHz; CDCl_3_; Me_4_Si) 172.0, 158.4, 154.0, 145.8, 133.6, 129.5, 129.4, 123.8, 122.6, 121.9, 119.9, 112.25, 108.6, 82.0, 66.5, 43.7, 28.1.

## 5. Conclusions

The findings of the knowledge gained in this and previous works demonstrate the flexibility of bivalent ligand design for constructing compounds whose ability to bind to the target biomolecule can be enhanced (switched on) or suppressed (switched off) by external stimuli. The conjugation of the pharmacophores of bisubstrate inhibitors of protein kinases via irradiation- and reduction-responsive cleavable linkers yielded compounds with strong ability to bind to the catalytic site of PKAcα (*K*_D_ values in one- to two-digit picomolar range). Cleavage of the linker in response to external stimuli (irradiation at 365 nm or chemical reduction, respectively) disassembled the compounds, which resulted in liberation of the active PKAcα. The deactivatable inhibitors of protein kinases described in this report could be valuable tools in applications which rely on the efficient temporal inhibition or capture of the catalytic subunits of basophilic protein kinases.

## Figures and Tables

**Table 1:** Inhibitory potencies (IC_50_) and the dissociation equilibrium constants (*K*_D_) of the characterized compounds towards PKAcα.

Compound	Assay ^1^	Parameter	Value ± SD/nM
ARC-1411	TGLI (A)	*K*_D_	0.003 ± 0.001; (0.003 [31])
H-89	TGLI (A), FA	*K*_D_	13 ± 3; (9 ± 1 [41])
	PhosphoSens^®^	IC_50_	670 ± 70
ARC-2121	TGLI (B)	*K*_D_	0.019 ± 0.007
ARC-2121, irradiated ^2^	TGLI (B)	*K*_D_^app^	0.6 ± 0.3
ARC-2194	TGLI (A)	*K*_D_	0.007 ± 0.001
	PhosphoSens^®^	IC_50_	2.9 ± 0.5
ARC-2194 + TCEP	FA	*K*_D_^app^	350 ± 30
	PhosphoSens^®^	IC_50_	48,000 ± 7000
**10**	FA	*K*_D_	650 ± 130
**11a:10** (1:1)	FA	*K*_D_^app^	190 ± 60
**11a**	FA	*K*_D_	460 ± 210
**2a**	FA	*K*_D_	550 ± 150

^1^ TGLI (A: 10 nM ARC-1063, 1 nM PKAcα; B: 10 nM ARC-1182, 1 nM PKAcα), FA (2 nM ARC-583, 3 nM PKAcα), PhosphoSens^®^ (1 mM MgATP, 10 µM peptide substrate, 0.5 nM PKAcα); ^2^ irradiated for 2 min under 365 nm LED.

## Data Availability

Please contact the corresponding author.

## Supplementary Materials

The following supporting information can be downloaded at: https://www.mdpi.com/article/10.3390/molecules27196689/s1, Figure S1: Simulated displacement binding curves; Figure S2: Custom-made LED array; Figure S3: Relative spectral distribution of 365 nm LEDs; Figure S4: HPLC analysis of ARC-2121 non-irradiated and irradiated; Figure S5: ESI-MS spectra of **11a** produced by irradiation of ARC-2121 for 120 s; Figure S6: ESI-MS spectra of **1a** and **2a** produced by reduction of ARC-2194; Figure S7: Structures of photoluminescence probes ARC-1182, ARC-1063, and ARC-583 and comparison compound H89; Table S1: Analytical data for novel compounds; S2: NMR spectra; S3: HPLC chromatograms and UV-Vis spectra of the main peaks of compounds **2a, 10, 11a**, ARC-2121, and ARC-2194.

## Appendix A

The reversible binding of the probe (ARC-583, ARC-1063, or ARC-1182) to PK is characterized by equilibrium constant $K_{D}^{*}$:

(A1) $$\mathrm{PK}+\mathrm{probe}\;⇌\;\mathrm{PK}:\mathrm{probe}\;K_{D}^{*}=\frac{\left[\mathrm{PK}\right]\left[\mathrm{probe}\right]}{\left[\mathrm{PK}:\mathrm{probe}\right]},\;$$

where [PK], [probe], and [PK:probe] correspond to equilibrium concentrations of PK, probe and PK:probe complex, respectively.

The reversible binding of the competing ligand/inhibitor to PK is characterized by equilibrium constant $K_{D}$:

(A2) $$\mathrm{PK}+I\;⇌\;\mathrm{PK}:I\;K_{D}=\frac{\left[\mathrm{PK}\right]\left[I\right]}{\left[\mathrm{PK}:I\right]},\;$$

The data corresponding to the measured anisotropy change of photoluminescent probe ARC-583 vs. log([I]_T_) were fitted to the following equation [40]:

(A3) $$r_{OBS}=\left(1-Z\right)\times \;r_{f}+Z\;\;r_{b},\;$$

where $r_{OBS}$ is the observed anisotropy, $r_{f}$ is the anisotropy of the free probe ARC-583, $r_{b}$ is the anisotropy of ARC-583:PK complex, and *Z* is a term that includes the fraction of the bound probe. Z takes into account the change of fluorescence intensity upon binding of ARC-583 to PK, as follows:

(A4) $$Z=\frac{Q\;\times \;F}{\left[1+F\left(Q-1\right)\right]},\;$$

where Q is the ratio of intensities (or quantum yields) of bound and free ARC-583 measured under the given assay conditions, and

(A5) $$F=\frac{\left[\mathrm{probe}:\mathrm{PK}\right]}{{\left[\mathrm{probe}\right]}_{T}},$$

where *F* is the fraction of the bound probe, [probe:PK] is the equilibrium concentration of the probe:PK complex, ${\left[\mathrm{probe}\right]}_{T}$ is the total concentration of the probe (whereas [probe]_T_ = [probe:PK] + [probe], where [probe] is the equilibrium concentration of the free probe), and the probe in the given case is ARC-583. If Q=1, Z=F.

The fraction of the bound probe *F* is related to the $K_{D}$, $K_{D}^{*}$, and total concentrations of the interacting components [I]_T_, [probe]_T_, and [PK]_T_ according to the following solution of the cubic equation [54]:

(A6) $$F=\frac{\left\{2\sqrt{a^{2}-3b}cos\frac{\theta }{3}-a\right\}}{3K_{D}^{*}+\left\{2\sqrt{a^{2}-3b}cos\frac{\theta }{3}-a\right\}},\;$$

where

(A7) $$\theta =arccos\frac{-2a^{3}+9ab-27c}{2\sqrt{{\left(a^{2}-3b\right)}^{3}}},\;$$

(A8) $$a=K_{D}+K_{D}^{*}+10^{X}+{\left[\mathrm{probe}\right]}_{T}-{\left[\mathrm{PK}\right]}_{T},\;$$

(A9) $$b=K_{D}\left({\left[\mathrm{probe}\right]}_{T}-{\left[\mathrm{PK}\right]}_{T}\right)+K_{D}^{*}\left(10^{X}-{\left[\mathrm{PK}\right]}_{T}\right)+K_{D}\times K_{D}^{*},\;$$

(A10) $$c=-K_{D}\times K_{D}^{*}\times {\left[E\right]}_{T},\;and$$

(A11) $$X=\log\left({\left[I\right]}_{T}\right).$$

The data corresponding to the measured TGLI of photoluminescent probe ARC-1063 or ARC-1182 vs. log([I]_T_) were fitted to the following equation [42]:

(A12) $$TGLI_{OBS}=TGLI_{0}+M\;\times \;F\;\times \;{\left[\mathrm{probe}\right]}_{T},\;$$

where *TGLI*_OBS_ is the observed TGLI signal, *TGLI*_0_ is the background TGLI signal, *M* is the molar TGLI signal of the probe:PK complex, and *F* is defined by Equation (A6), where the probe is either ARC-1063 or ARC-1182.
