# Supplementary material for: Deactivatable Bisubstrate Inhibitors of Protein Kinases
Source: Molecules. 2022 Oct 8;27(19):6689. doi: 10.3390/molecules27196689 (PMC9573699; doi:10.3390/molecules27196689)

Deactivatable Bisubstrate Inhibitors of Protein Kinases

Tanel Sõrmus, Darja Lavogina, Erki Enkvist, Asko Uri and Kaido Viht*

Institute of Chemistry, University of Tartu, 14A Ravila St., 50411 Tartu, Estonia; tanel.sormus@ut.ee (T.S.); darja.lavogina@ut.ee (D.L.); erki.enkvist@ut.ee (E.E.); asko.uri@ut.ee (A.U.). *Correspondence: kaido.viht@ut.ee; Tel.: +372-737-5250

Electronic Supplementary Information

Contents

S1. Supplementary schemes, figures, and tables 2

S2. NMR spectra 9

S2.1 Compound 5; ^{1}H and ^{13}C spectra. 9

S2.2 Compound 6; ^{1}H and ^{13}C spectra. 11

S2.3 Compound 7; ^{1}H spectra. 13

S2.4 Compound 3; ^{1}H spectra. 14

S6.5 Compound 8; ^{1}H spectra. 15

S3. HPLC chromatograms and UV-Vis spectra of the main peaks of compounds 2a, 10, 11a, ARC-2121, and ARC-2194 17

S3.1 Compound 2a 17

S3.2 Compound 10 18

S3.3 Compound 11a 19

S3.4 ARC-2121 20

S3.5 ARC-2194 21

S1. Supplementary schemes, figures, and tables

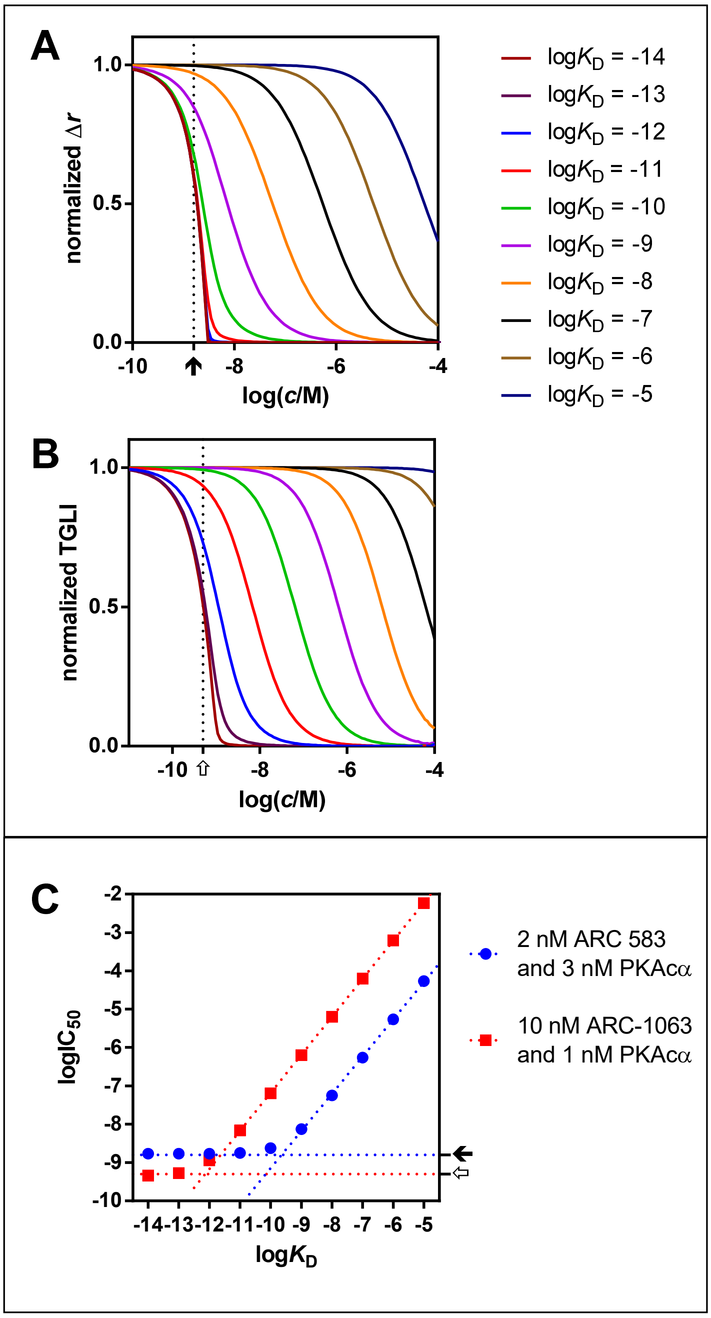

Figure S1. Simulated displacement binding curves. (**A**) and (**B**) Theoretical displacement curves simulated by Graphpad Prism software (v 6.04) for compounds covering the range of K_D values from 10 fM to 10 µM (specified in Figure legend). (**A**) The curves correspond to the **Equation (A3)** (in Appendix A) with the following parameters: K_D^*(ARC-583:PKAcα) = 0.5 nM, $[\text{PKAc}\alpha]_T$ = 3 nM, $[\text{ARC-583}]_T$ = 2 nM, and Q = 0.8. (**B**) The curves correspond to the **Equation (A12)** (in Appendix A) with the following parameters: K_D^*(ARC-Lum(Fluo):PKAcα) = 15 pM, $[\text{PKAc}\alpha]_T$ = 1 nM, and $[\text{ARC-Lum(Fluo)}]_T$ = 10 nM, where ARC-Lum(Fluo) corresponds to either ARC-1063 or ARC-1182. (**C**) The displacement curves from (A) and (B) were analyzed by sigmoidal dose-response model and the corresponding $\log IC_{50}$ values were plotted as funtions of $\log K_D$ values of the corresponding displacement curves. (**A-C**) The tight binding limits of IC_{50} values at the corresponding assay conditions are indicated by white and black arrows.

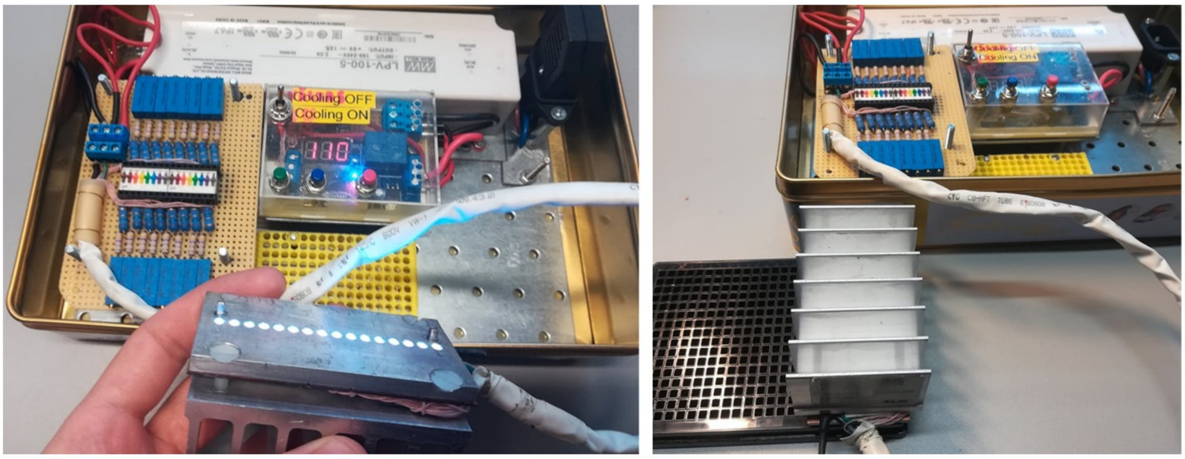

Figure S2. Photos of the custom-made LED array. The timer-equipped custom-made LED array accommodating 16 LEDs [150 mcd, λ_{max} = 365 nm, 975 mW, part number LTPL-C034UVH385] designed for irradiation of one row of wells at a time in a 384-well microtiter plate.

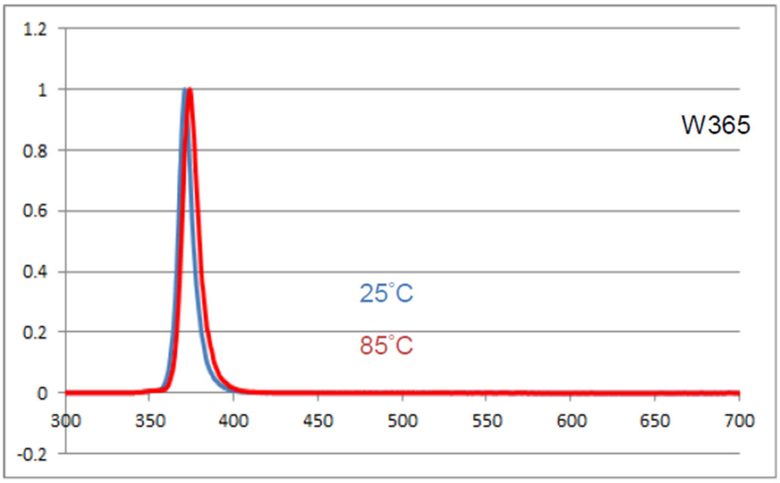

Figure S3. Relative spectral distribution of 365 nm LEDs. The blue and red lines correspond to the spectral distribution of LEDs at 25 °C and 85 °C, respectively (from manufacturer's datasheet).

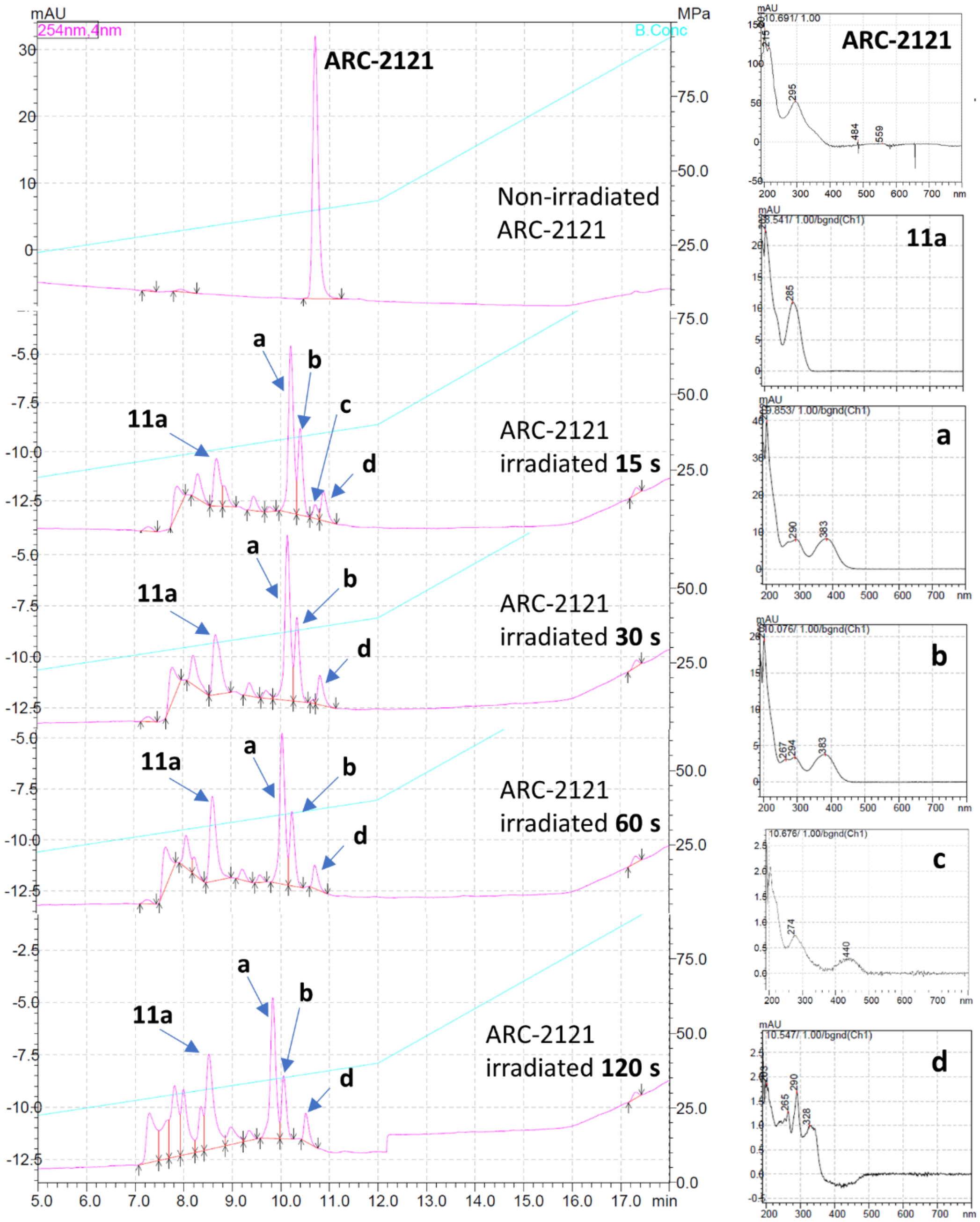

Figure S4. HPLC analysis of ARC-2121 non-irradiated and irradiated. ARC-2121 was irradiated for various durations (15 s, 30 s, 60 s, 120 s) using the custom-made LED array at 365 nm. (**a**)-(**d**) UV-Vis spectra of the peaks corresponding to the unidentified photolysis products of ARC-2121.

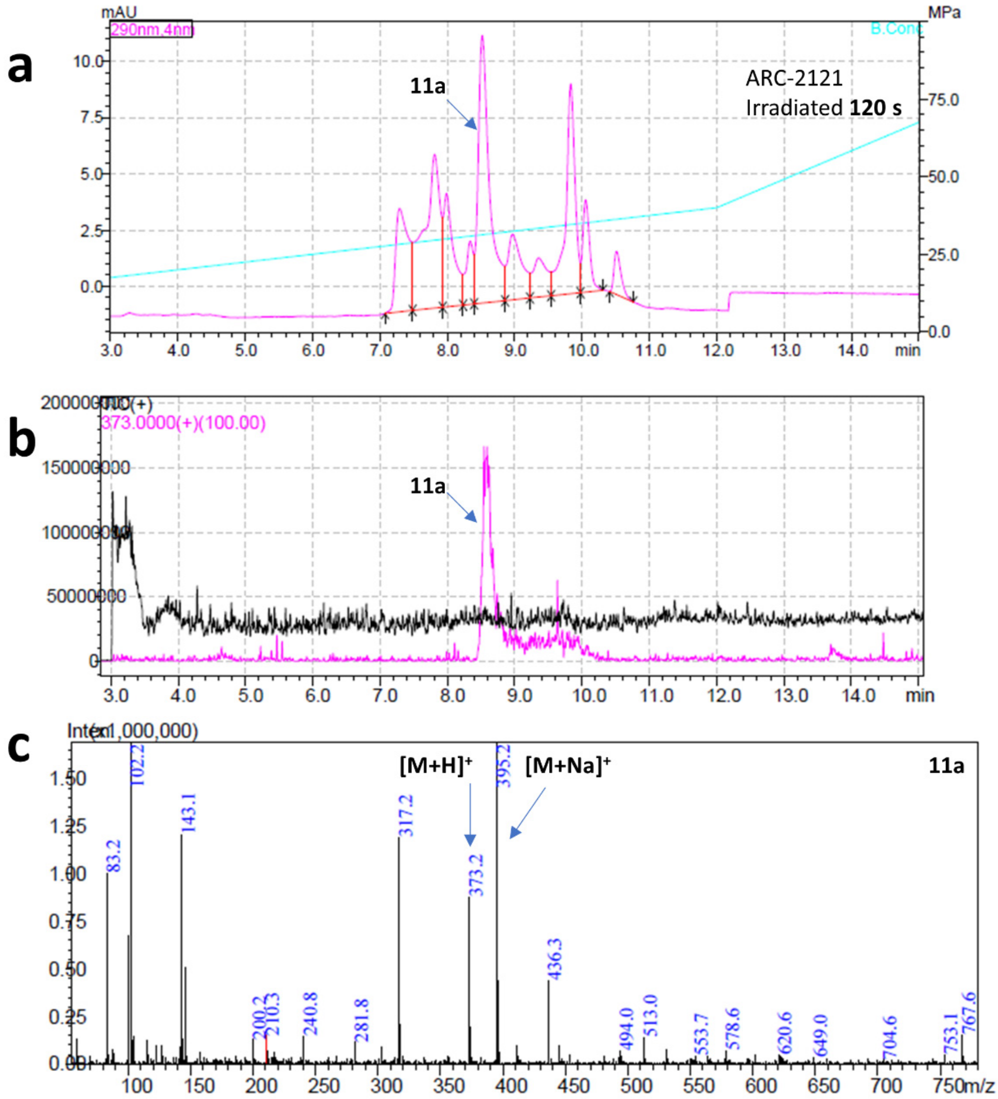

Figure S5. ESI-MS spectra of **11a** produced by irradiation of ARC-2121 for 120 s. (**a**) HPLC chromatogram; (**b**) and (**c**) ESI-MS spectra of **11a**.

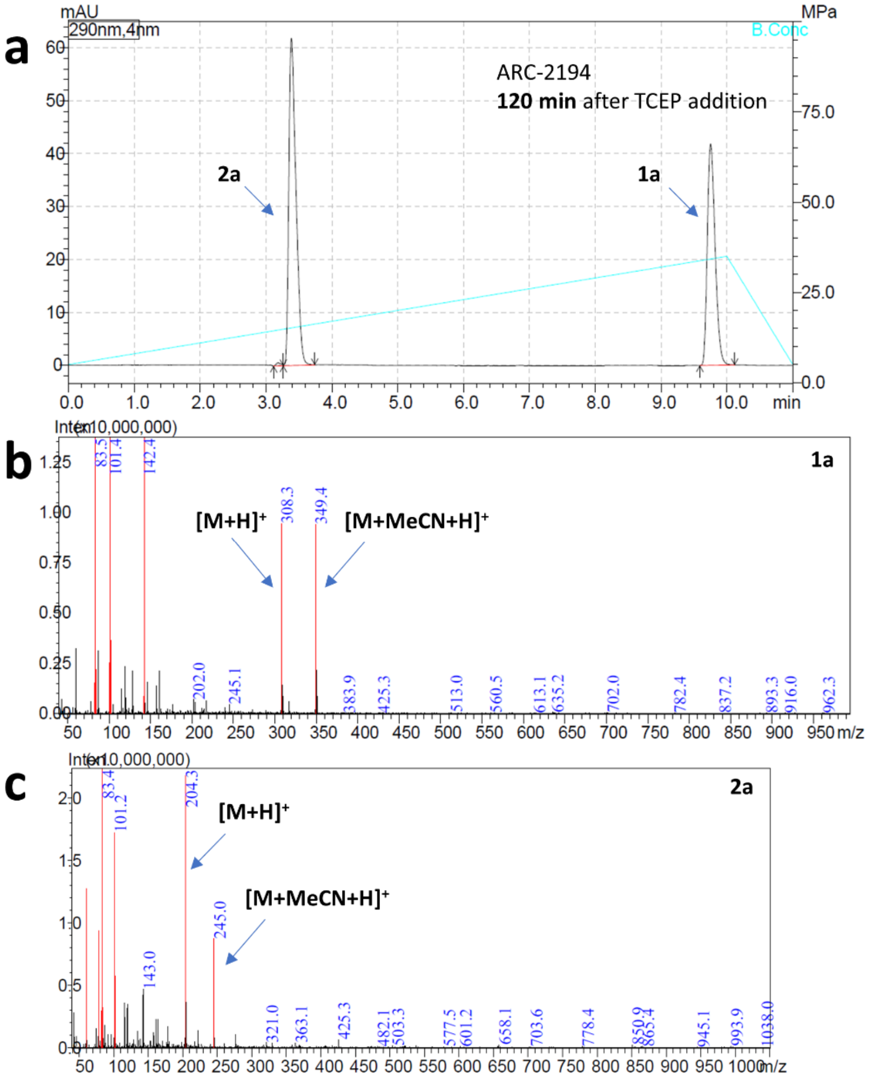

Figure S6. ESI-MS spectra of **1a** and **2a** produced by reduction of ARC-2194. (**a**) HPLC chromatogram; (**b**) **1a** and (**c**) **2a** ESI-MS spectra.

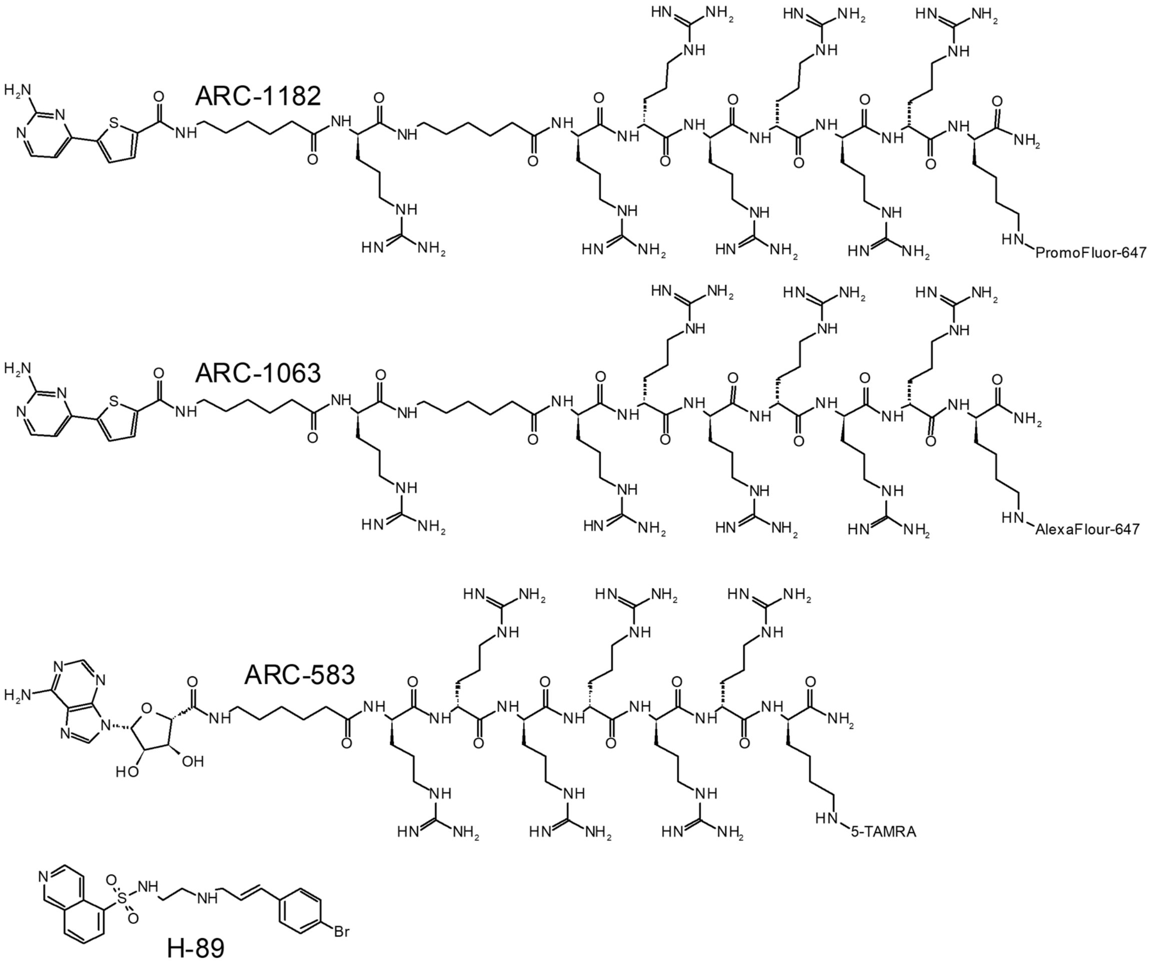

Figure S7. Structures of photoluminescent probes ARC-1182, ARC-1063, and ARC-583 and reference compound H89.

Table S1. Analytical data for novel compounds **10**, **11a**, ARC-2121, and ARC-2194

Compound code	Molecular formula	MW (neutral form)	MS Data *M/z*	HPLC data	
				t_R / min	Purity (HPLC peak area % at 254 nm)
**10**	$C_{51}H_{82}N_{26}O_{11}$	1235.37	$[M+2H]^{2+}$ calculated for $C_{51}H_{84}N_{26}O_{11}^{2+}$ 618.3401; found: 618.3401[a]	7.7[d]	96.5
**11a**	$C_{19}H_{28}N_6O_2$	372.47	$[M+H]^{+}$ calculated for $C_{19}H_{29}N_6O_2^{+}$ 373.2347; found: 373.2347[a]	7.8[d]	98.9
ARC-2121	$C_{70}H_{110}N_{32}O_{12}$	1591.83	$[M+2H+TFA]^{2+}$calculated for $C_{72}H_{112}N_{32}O_{14}F_3^{2+}$ 853.5; found: 853.7[b]	10.7[c]	98.0
ARC-2194	$C_{52}H_{94}N_{30}O_{10}S_2$	1363.63	$[M+3H+2TFA]^{3+}$ calculated for $C_{56}H_{99}N_{30}O_{14}S_2F_6^{3+}$ 531.2429; found: 531.2418[a]	6.2[d]	97.3

[a] ESI HRMS; [b] ESI MS; [c]0.1% TFA/(10 – 40% MeCN/H_2O in 12 min); [d]0.1% TFA/(10 – 70% MeCN/H_2O in 20 min)

S2. NMR spectra

S2.1 Compound 5; ^{1}H and ^{13}C spectra.

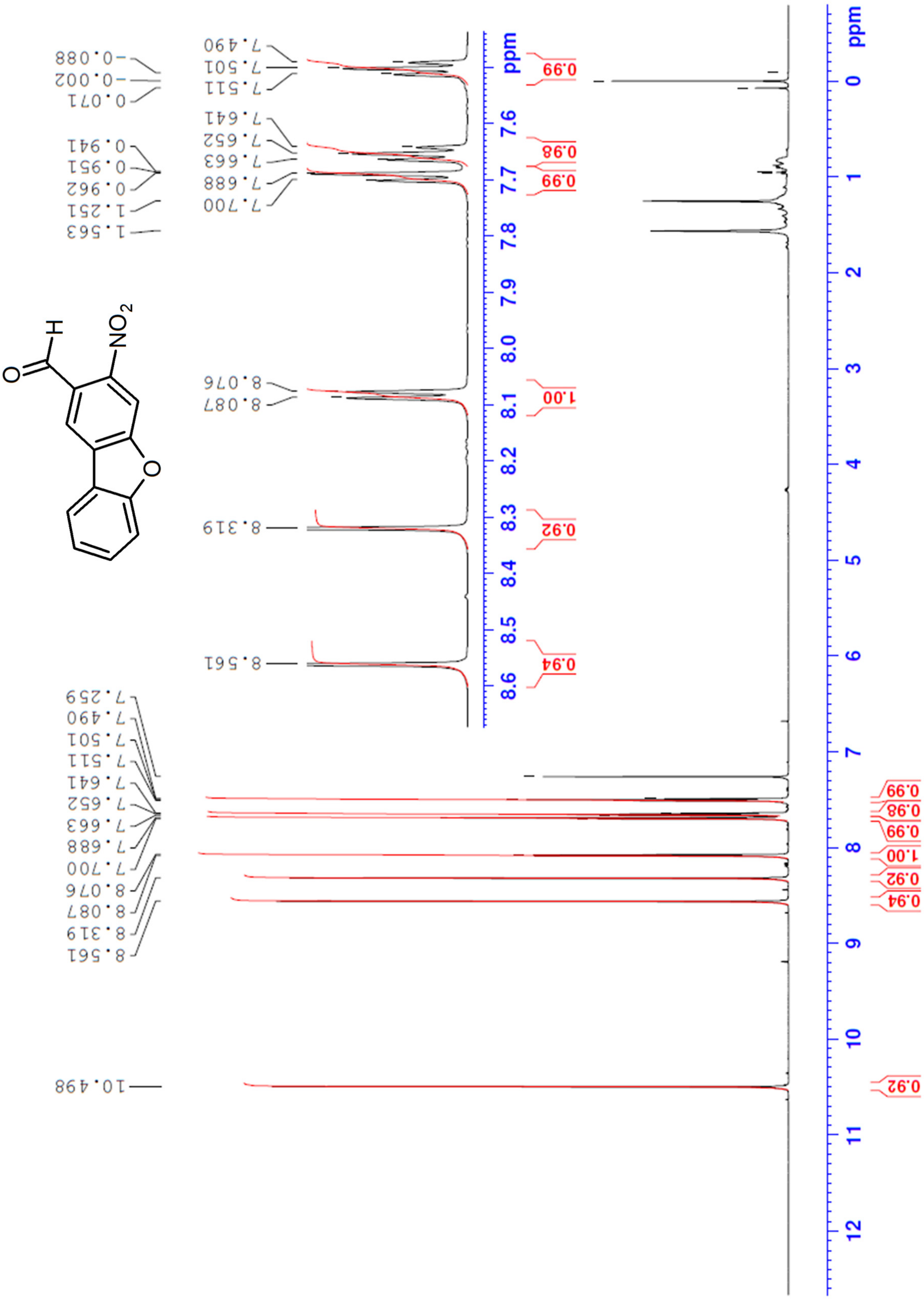

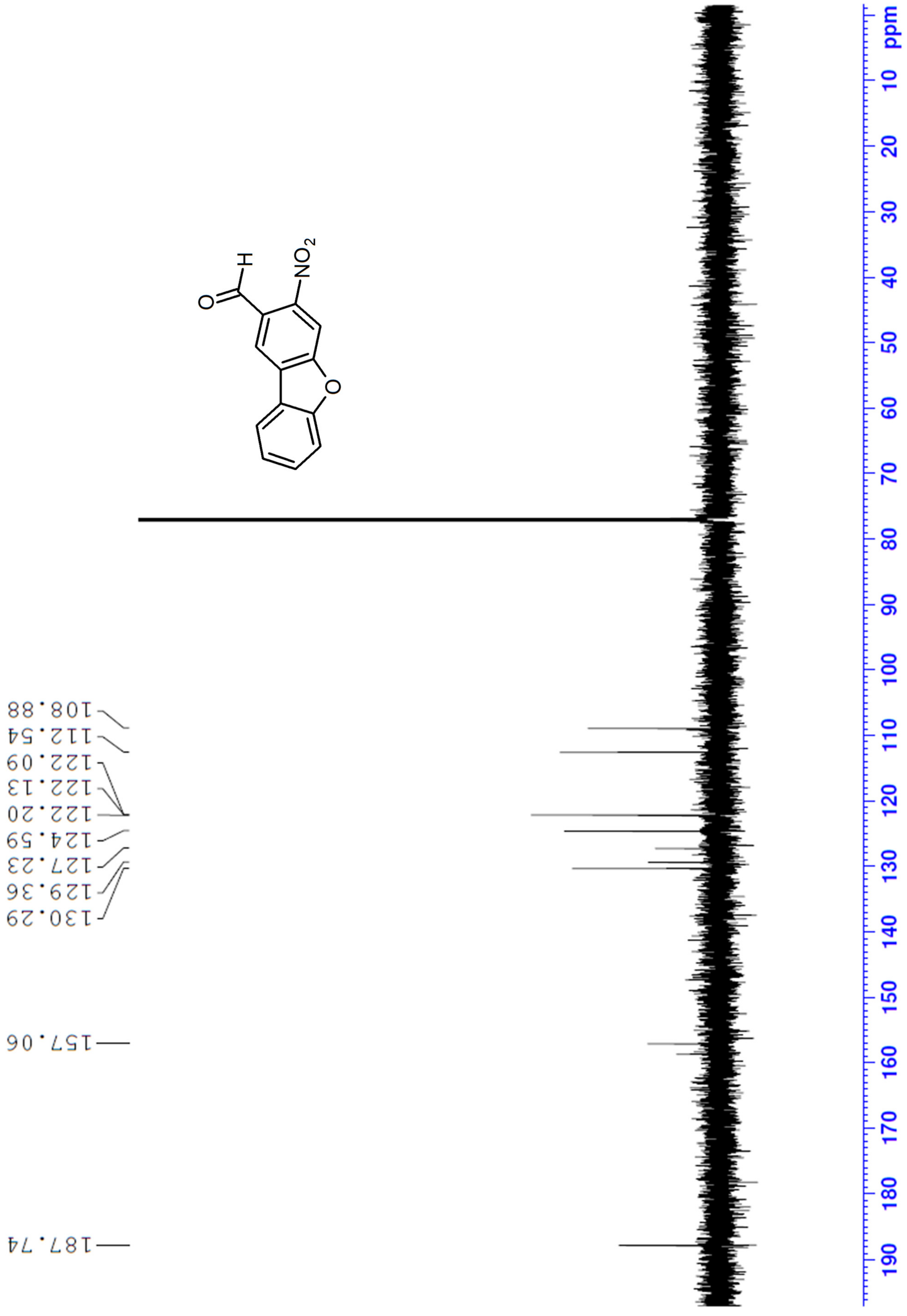

108.88
112.54
122.09
122.13
122.20
124.59
127.23
129.36
130.29
157.06
187.74
ppm

S2.2 Compound ***6****; ^{1}H and ^{13}C spectra.*

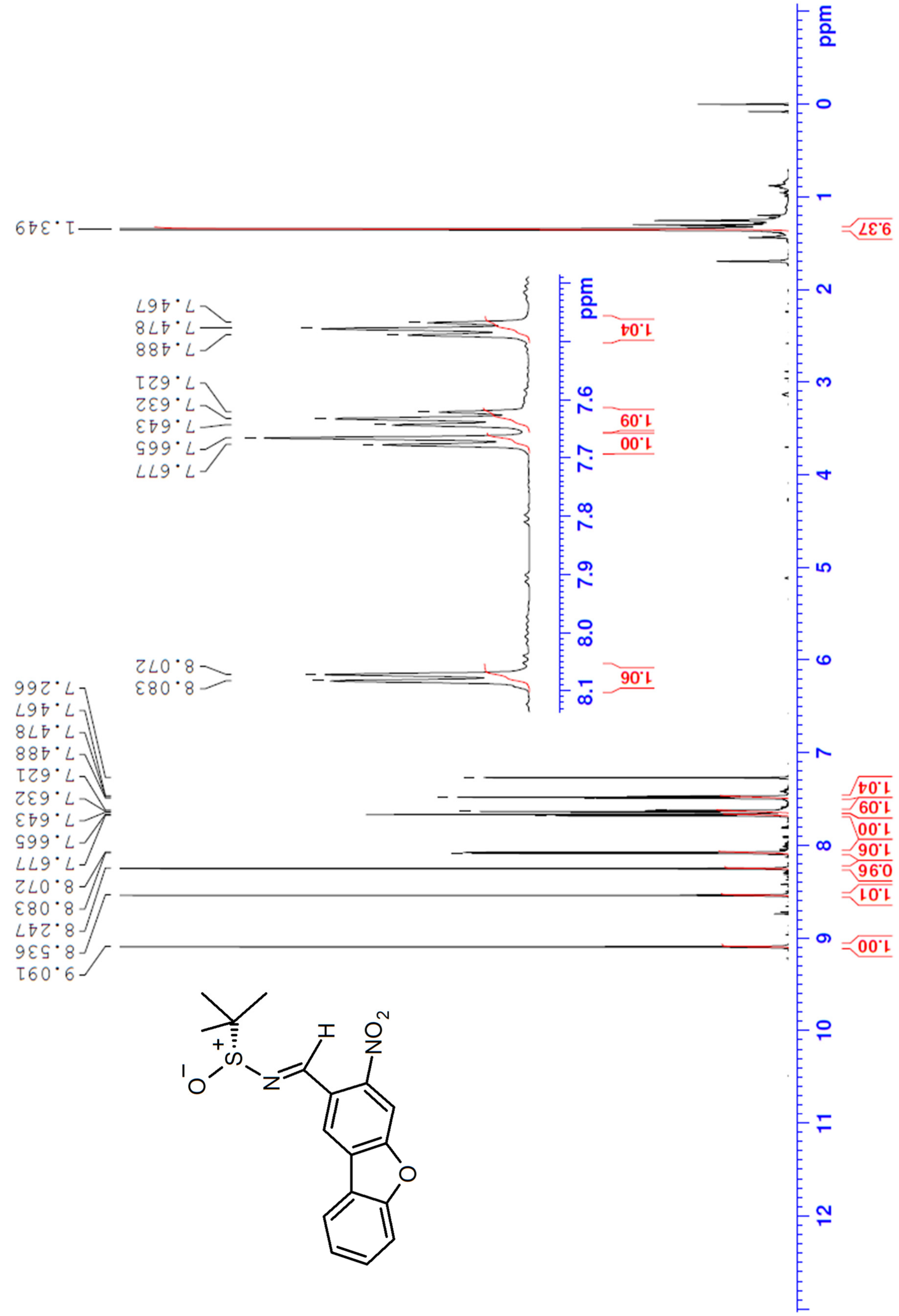

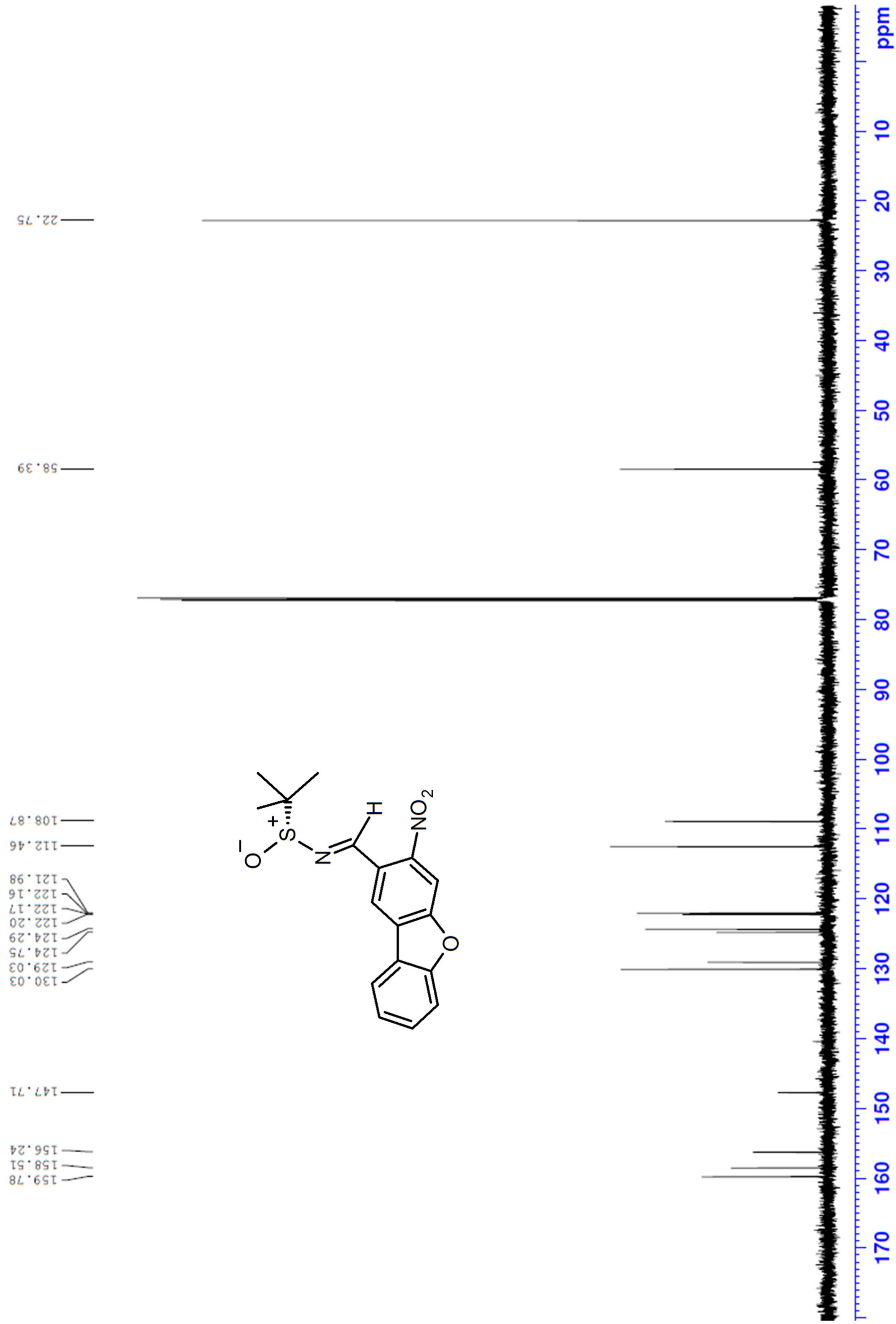
22.75
58.39
108.87
112.46
121.98
122.16
122.17
122.20
124.29
124.75
129.03
130.03
147.71
156.24
158.51
159.78
ppm
10
20
30
40
50
60
70
80
90
100
110
120
130
140
150
160
170
H
NO_2
N
S
O

S2.3 Compound 7; ^{1}H spectra.

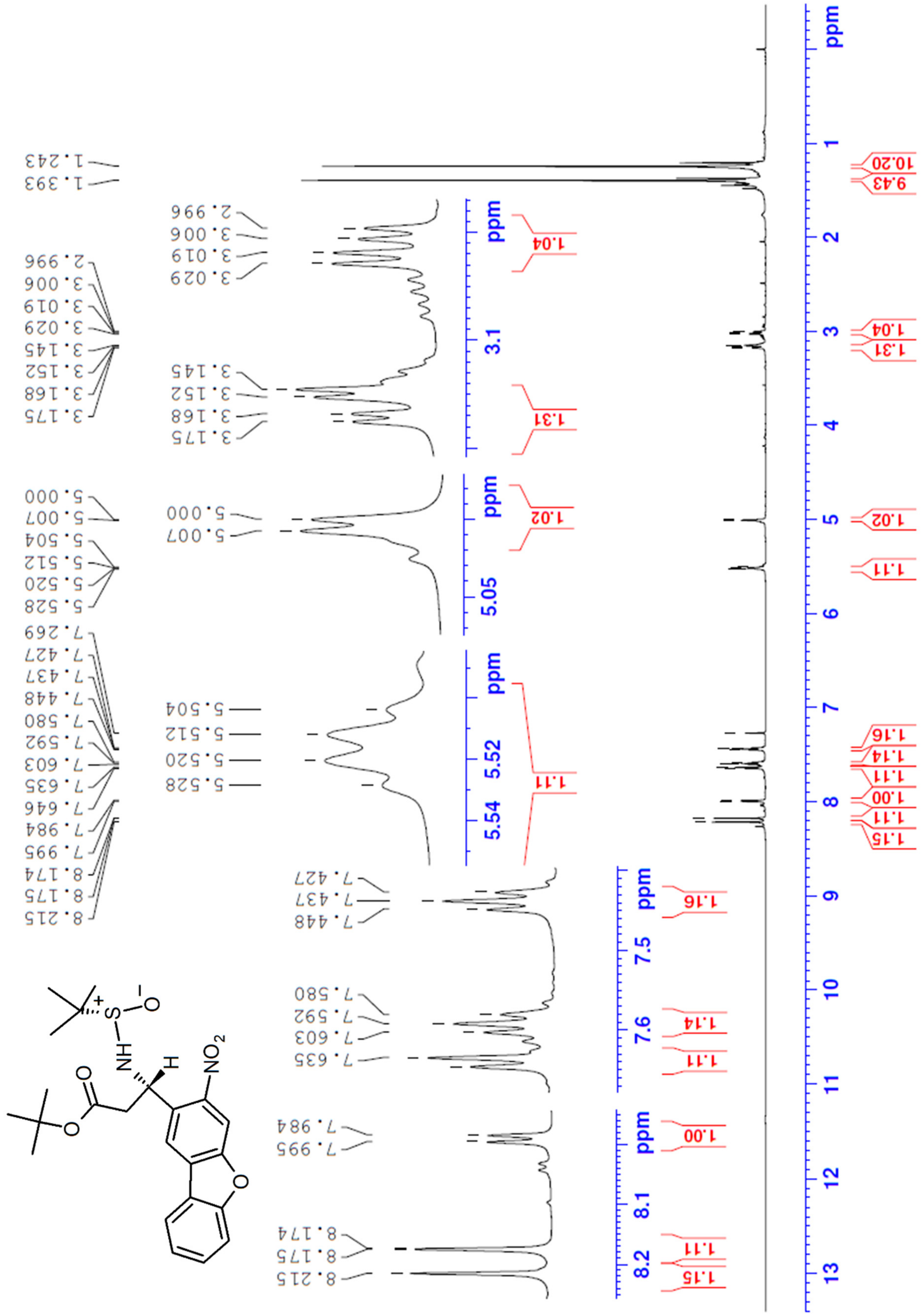

S2.4 Compound 3; ^{1}H spectra.

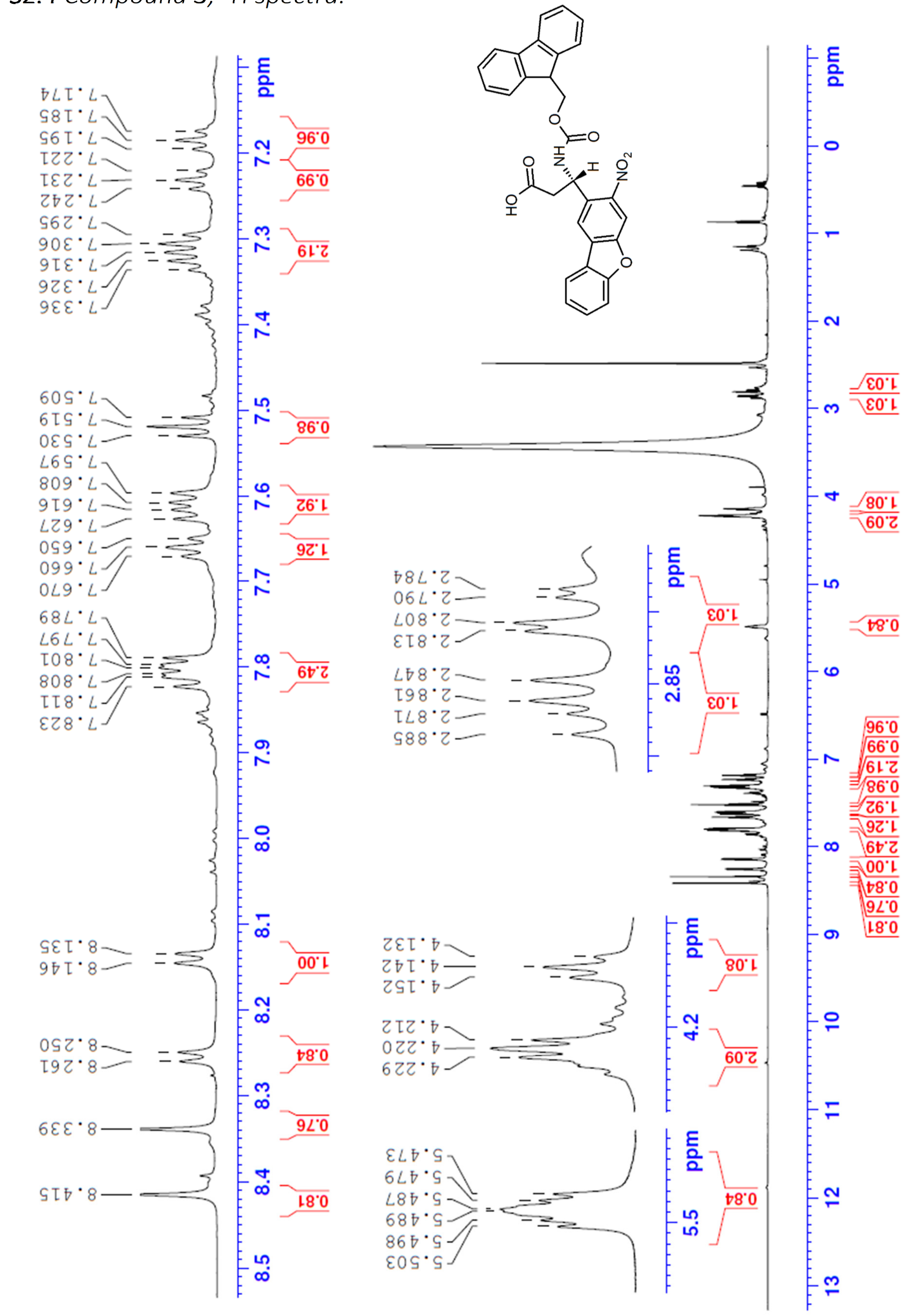

S6.5 Compound 8; ¹H spectra.

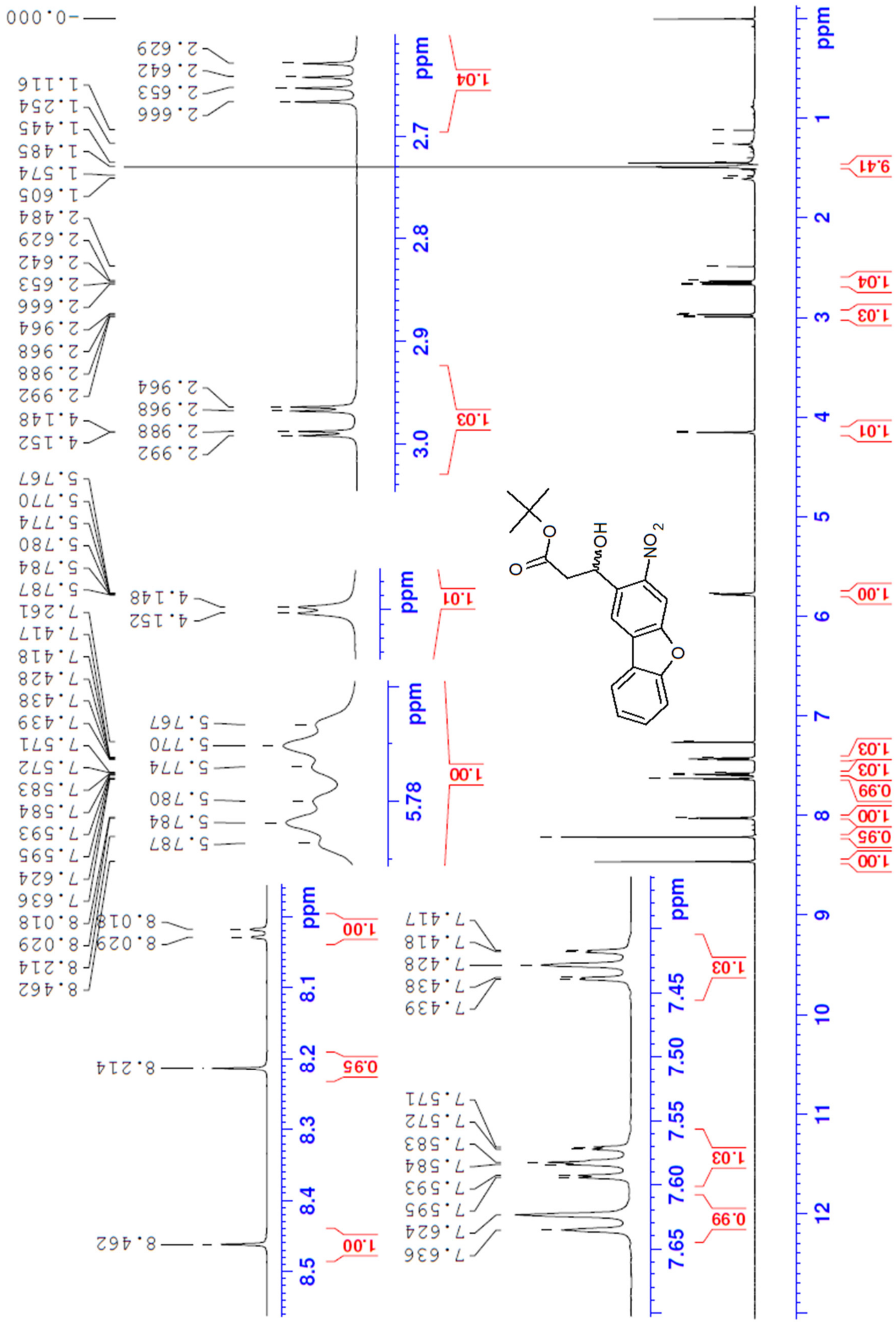

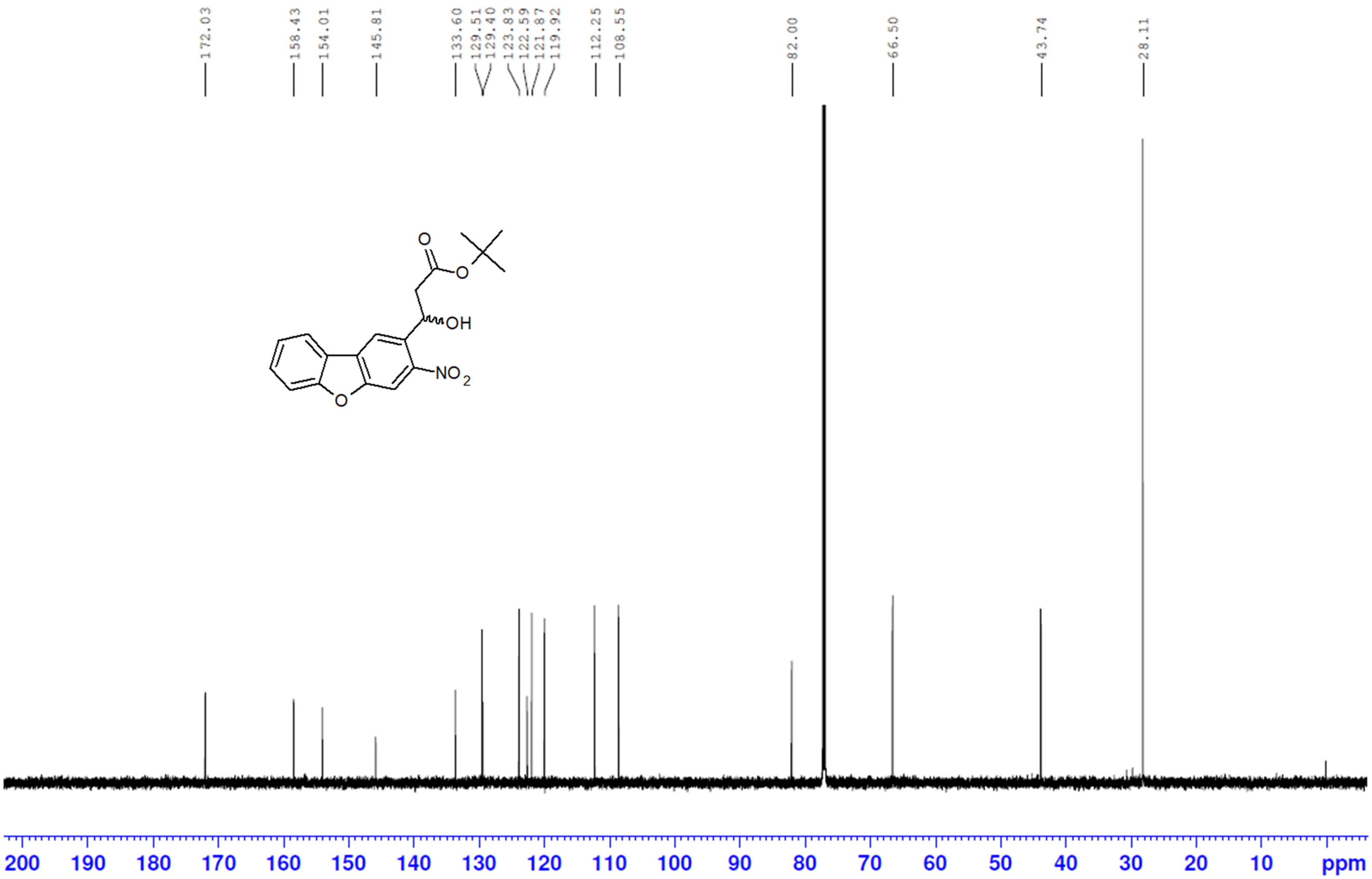
172.03
158.43
154.01
145.81
133.60
129.51
129.40
123.83
122.59
121.87
119.92
112.25
108.55
82.00
66.50
43.74
28.11
OH
NO2
O
O
O
200
190
180
170
160
150
140
130
120
110
100
90
80
70
60
50
40
30
20
10
ppm

S3. HPLC chromatograms and UV-Vis spectra of the main peaks of compounds 2a, 10, 11a, ARC-2121, and ARC-2194

S3.1 Compound 2a

Gradient: 0.1% TFA/(5 – 75% MeCN/H_2O in 20 min)

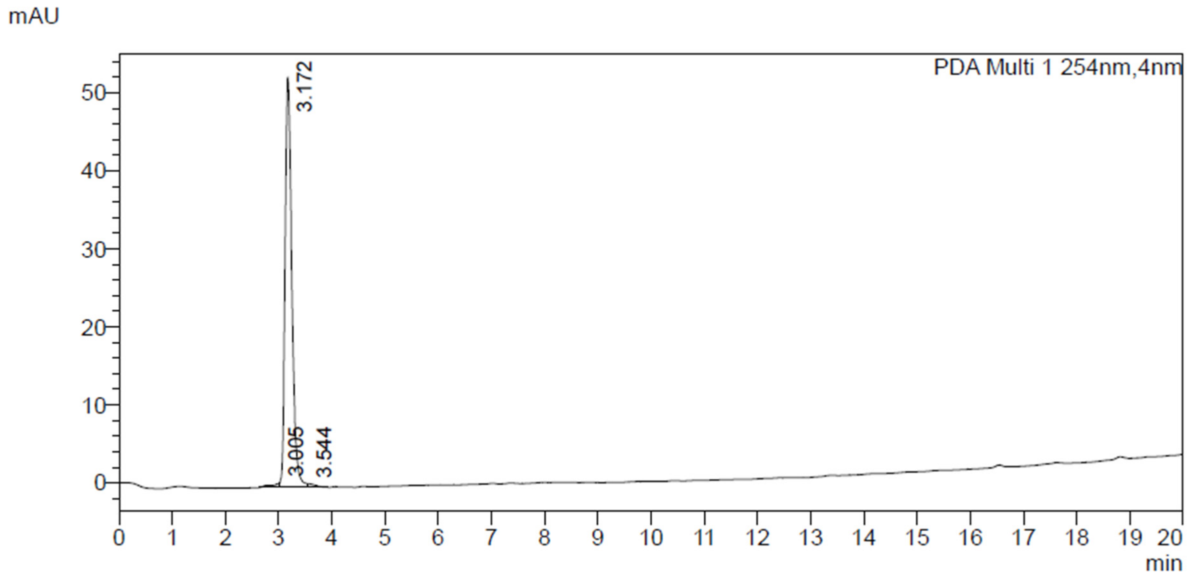

Peak Table

PDA Ch1 254nm

Peak#	Ret. Time	Area	Area%
1	3.005	5622	1.187
2	3.172	463579	97.861
3	3.544	4512	0.953
Total		473714	100.000

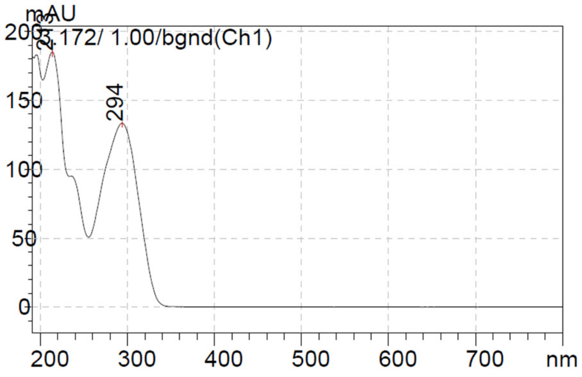

S3.2 Compound 10

Gradient: 0.1% TFA/(10 – 70% MeCN/H_2O in 20 min)

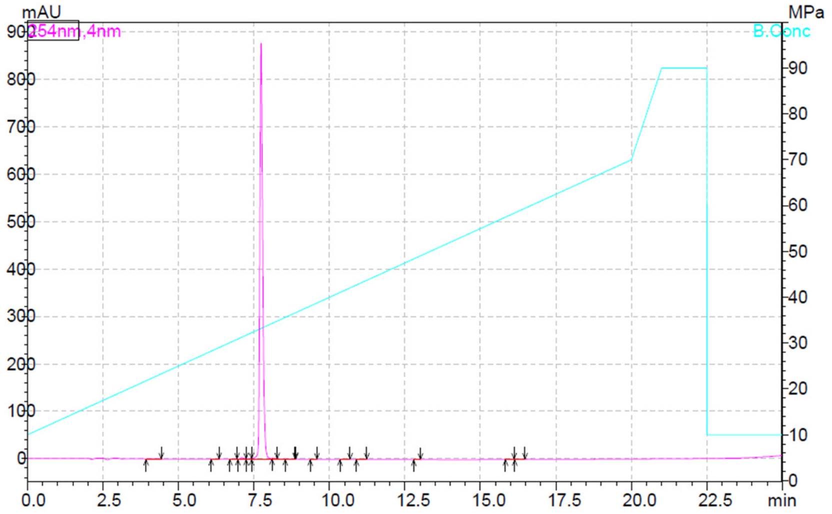

Peak Table

PDA Ch1 254nm

Peak#	Ret. Time	Area	Area%
1	1.932	17242	0.288
2	2.430	60827	1.015
3	2.926	45866	0.765
4	4.048	11216	0.187
5	6.178	1157	0.019
6	6.810	1401	0.023
7	7.094	24306	0.405
8	7.343	8964	0.150
9	7.735	5783558	96.473
10	8.142	1446	0.024
11	8.702	1946	0.032
12	9.456	1586	0.026
13	10.470	4193	0.070
14	11.028	4244	0.071
15	11.638	1905	0.032
16	12.886	1053	0.018
17	13.180	3365	0.056
18	15.135	1623	0.027
19	15.941	2754	0.046
20	16.290	6733	0.112
21	16.607	7626	0.127
22	17.351	2000	0.033
Total		5995011	100.000

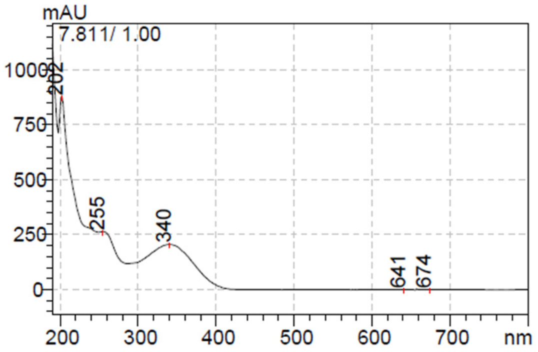

S3.3 Compound ***11a***

Gradient: 0.1% TFA/(10 – 70% MeCN/H_2O in 20 min)

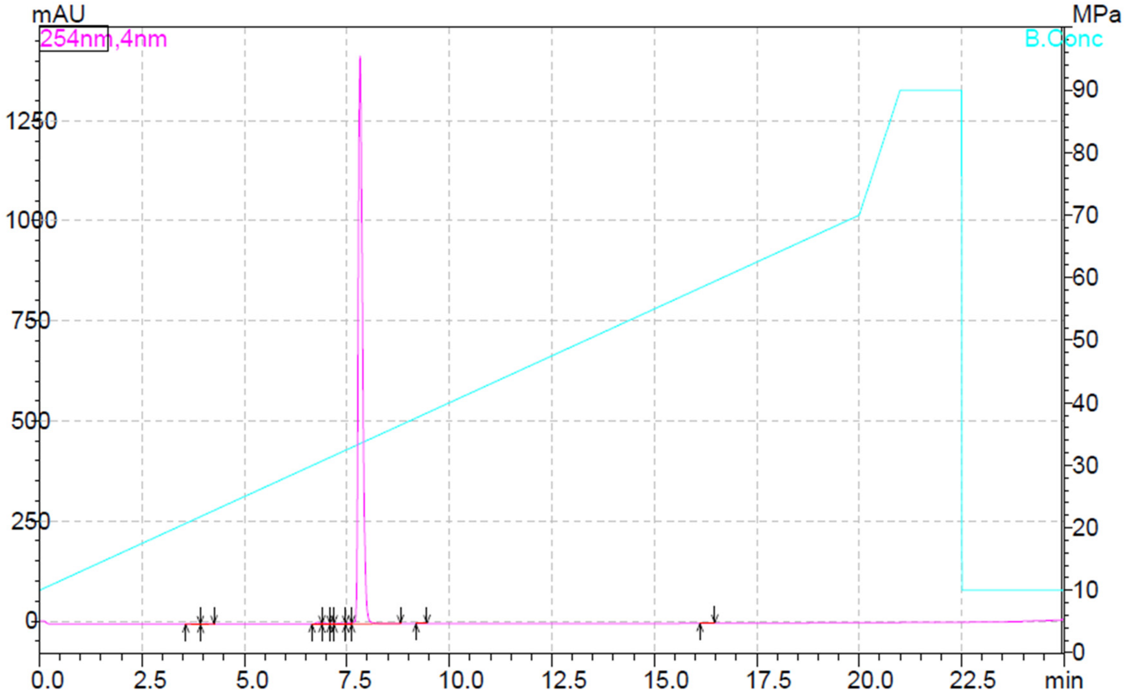

Peak Table

PDA Ch1 254nm

Peak#	Ret. Time	Area	Area%
1	3.696	10682	0.103
2	4.064	13142	0.127
3	6.781	25510	0.246
4	6.964	13874	0.134
5	7.125	2621	0.025
6	7.318	18223	0.176
7	7.573	18596	0.179
8	7.820	10273470	98.943
9	9.308	1678	0.016
10	16.276	5415	0.052
Total		10383211	100.000

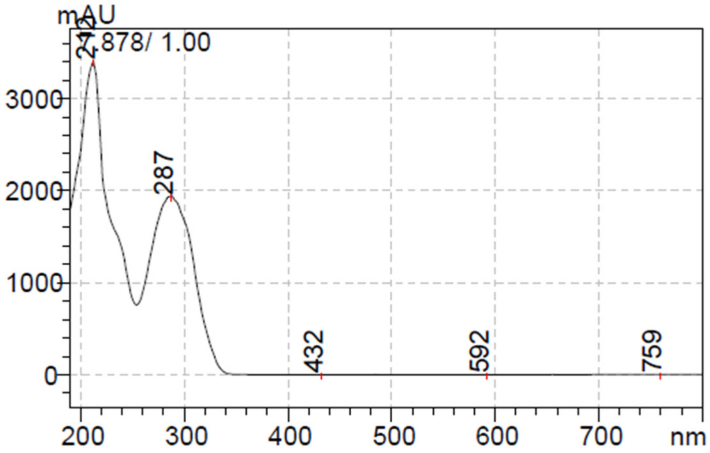

S3.4 ARC-2121

Gradient: 0.1% TFA/(10 – 40% MeCN/H_2O in 12 min)

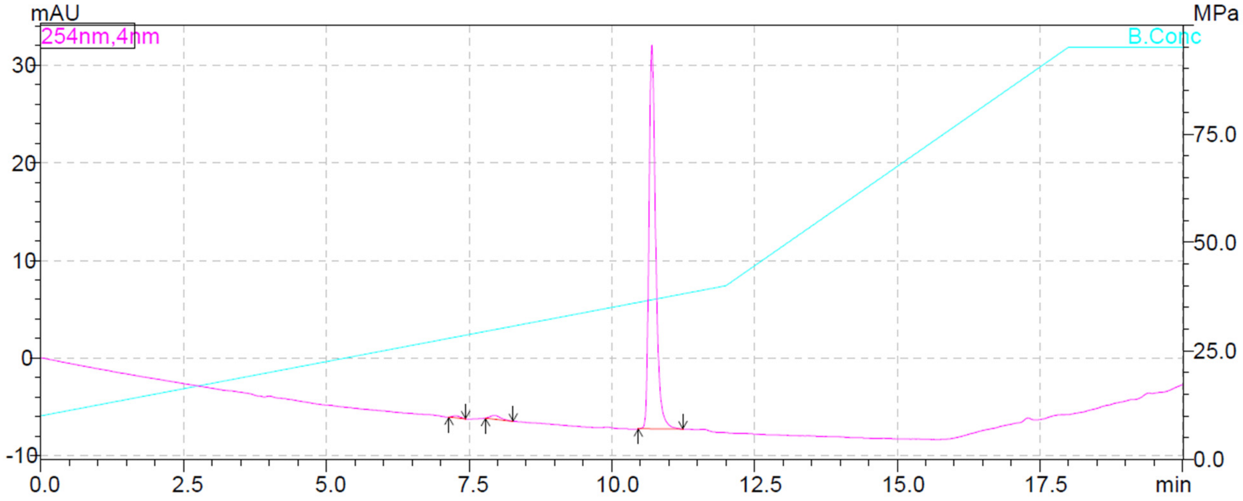

Peak Table

PDA Ch1 254nm

Peak#	Ret. Time	Area	Area%
1	7.253	1912	0.581
2	7.952	4708	1.430
3	10.702	322612	97.989
Total		329233	100.000

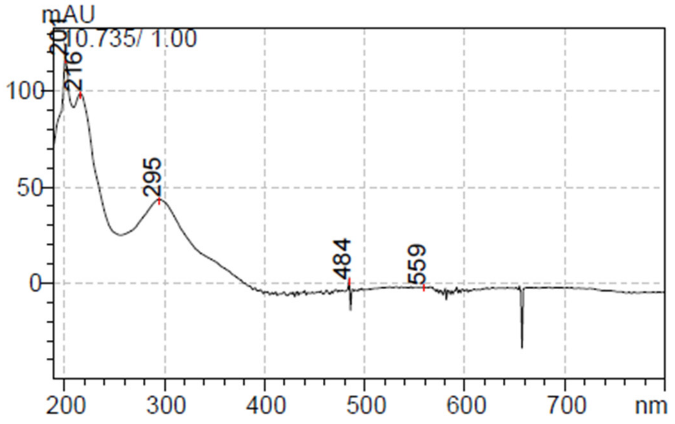

S3.5 ARC-2194

Gradient: 0.1% TFA/(10 – 70% MeCN/H_2O in 20 min)

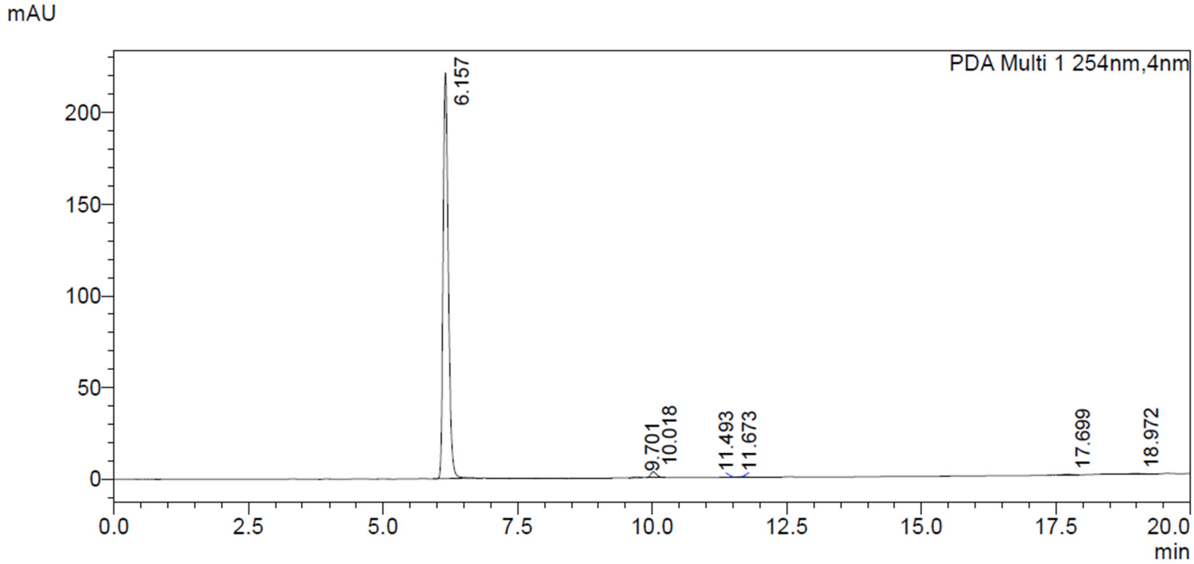

Peak Table

PDA Ch1 254nm

Peak#	Ret. Time	Area	Area%
1	6.157	1466434	97.285
2	9.701	2943	0.195
3	10.018	24522	1.627
4	11.493	1582	0.105
5	11.673	3930	0.261
6	17.699	3674	0.244
7	18.972	4273	0.283
Total		1507358	100.000

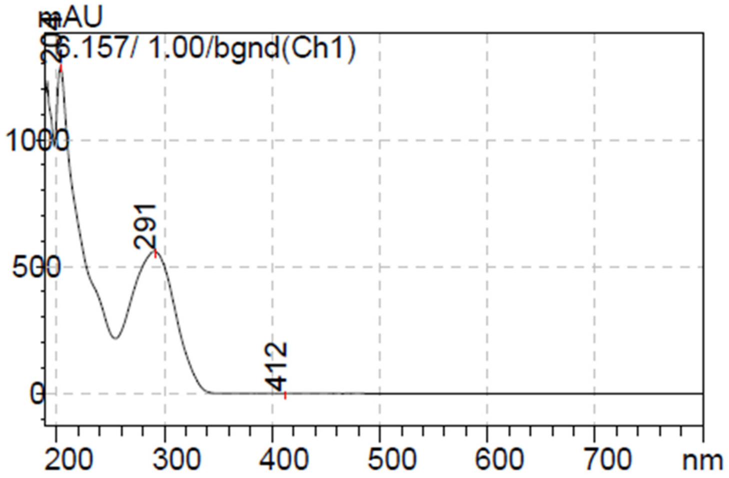

Supplement: Supplementary file 1 [file molecules-27-06689-s001.zip › molecules-1917405-supplementary.pdf]
